# Supplementary material for: Policies for Easing COVID-19 Pandemic Travel Restrictions
Source: medRxiv. 2021 Apr 20:2021.04.14.21255465. Preprint. [Version 1] doi: 10.1101/2021.04.14.21255465 (PMC8077591; doi:10.1101/2021.04.14.21255465)
Supplement: 1 [file NIHPP2021.04.14.21255465-supplement-1.pdf]

medRxiv preprint doi: https://doi.org/10.1101/2021.04.14.21255465; this version posted April 20, 2021. The copyright holder for this preprint (which was not certified by peer review) is the author/funder, who has granted medRxiv a license to display the preprint in perpetuity.
It is made available under a CC-BY 4.0 International license .

Supplementary Materials: Policies for Easing COVID-19 Pandemic Travel Restrictions

Thien-Minh Le[1*], Louis Raynal[1], Octavious Talbot[1], Hali Hambridge[1], Christopher Drovandi[3], Antonietta Mira[2], Kerrie Mengersen[3], Jukka-Pekka Onnela[1*]

[1]Department of Biostatistics, Harvard T.H. Chan School of Public Health, Boston, Massachusetts, U.S.A.

[2] Institute of Computational Science, Universita della Svizzera Italiana, Lugano, Switzerland

[3] School of Mathematical Sciences, Science and Engineering Faculty, Queensland University Technology, Brisbane, Australia

[*]To whom correspondence should be addressed: thle@hsph.harvard.edu & onnela@hsph.harvard.edu.

The supplementary materials include:

Models and methods

Simulation studies

Figure S1

Tables S1-S7

References

medRxiv preprint doi: https://doi.org/10.1101/2021.04.14.21255465; this version posted April 20, 2021. The copyright holder for this preprint (which was not certified by peer review) is the author/funder, who has granted medRxiv a license to display the preprint in perpetuity.
It is made available under a CC-BY 4.0 International license .

1 Models and methods

1.1 Local travel model

We first consider a local epidemiological model as in Warne et al. (2020) (*1*). In this local model, for each country, at a given time, its population's status is divided into 6 mutually exclusive compartments: susceptible (S), undetected infected (I), active confirmed (A), confirmed recovered (R), confirmed deceased (D) and unconfirmed recovered (R^u). Its dynamic states evolve as:

$$S \xrightarrow{\alpha} I, \qquad I \xrightarrow{\gamma} A, \qquad A \xrightarrow{\beta} R, \qquad A \xrightarrow{\delta} D, \qquad I \xrightarrow{\beta} R^u,$$

where α is the transmission rate, γ is the identification rate, β is the recovery rate, and δ is the death rate. Suppose that for a given country the status of its population at time t is $\mathbf{X}(t) = [S(t), I(t), A(t), R(t), D(t), R^u(t)]$, and $\theta = (\alpha, \beta, \delta, \gamma)$ represents the parameter of the statistical model for the country. Using the tau leaping method by Gillespie (2001) (*2*), the status of its population at time $(t+\tau)$ evolves as $\mathbf{X}(t+\tau) = \mathbf{X}(t) + \sum_{i=1}^{5} Y_j\big(h_j(\mathbf{X}(t))\tau\big)\nu_i$. In the above formula, ν_i, $i = 1, \cdots, 5$, are the transition vectors, $\nu_1 = [-1, 1, 0, 0, 0, 0]^T$, $\nu_2 = [0, -1, 1, 0, 0, 0]^T$, $\nu_3 = [0, 0, -1, 1, 0, 0]^T$, $\nu_4 = [0, 0, -1, 0, 1, 0]^T$, and $\nu_5 = [0, -1, 0, 0, 0, 1]^T$. Let the random variables $Y_i\big(h_i(\mathbf{X}(t))\tau\big)$ be Poisson distributed with rates $h_i(\mathbf{X}(t)\tau)$, for $i \in \{1, \cdots, 5\}$. More specifically, $h_1(\mathbf{X}(t)\tau) = \alpha\tau\frac{S(t)I(t)}{P}$, $h_2(\mathbf{X}(t)) = \gamma\tau I(t)$, $h_3(\mathbf{X}(t)) = \beta\tau A(t)$, $h_4(\mathbf{X}(t)) = \delta\tau A(t)$, $h_5(\mathbf{X}(t)) = \beta\tau I(t)$, and P is the country's population.

We choose $\tau = 1$, which represents the change in population status after each day. Then the dynamic evolution of the epidemic in the country can be elaborated further as follows. After each day, the state of the model evolves from $\mathbf{X}(t) = [S(t), I(t), A(t), R(t), D(t), R^u(t)]$ to $\mathbf{X}(t+1) = [S(t+1), I(t+1), A(t+1), R(t+1), D(t+1), R^u(t+1)]$ by the transformation $\mathbf{X}(t+1) = \mathbf{X}(t) + \sum_{j=1}^{5} Y_j\big(h_j(\mathbf{X}(t))\big)\nu_j$. In particular, $S(t+1) = S(t) - Y_1^t$, $I(t+1) = I(t)+Y_1^t-Y_2^t-Y_5^t$, $A(t+1) = A(t)+Y_2^t-Y_3^t-Y_4^t$, $R(t+1) = R(t)+Y_3^t$, $D(1) = D(t)+Y_4^t$,

medRxiv preprint doi: https://doi.org/10.1101/2021.04.14.21255465; this version posted April 20, 2021. The copyright holder for this preprint (which was not certified by peer review) is the author/funder, who has granted medRxiv a license to display the preprint in perpetuity.
It is made available under a CC-BY 4.0 International license .

$R^u(t+1) = R^u(t) + Y_5^t$, where Y_i^t are Poisson distributed with rates $h_i(\mathbf{X}(t))$, $i = 1, \cdots, 5$, $h_1(\mathbf{X}(t)) = \alpha\frac{S(t)I(t)}{P}$, $h_2(\mathbf{X}(t)) = \gamma I(t)$, $h_3(\mathbf{X}(t)) = \beta A(t)$, $h_4(\mathbf{X}(t)) = \delta A(t)$, $h_5(\mathbf{X}(t)) = \beta I(t)$.

Notice that the local model can be made more flexible by letting the transmission rate α change over time, i.e., setting $\alpha = \alpha_1 \mathcal{I}_{0,T(1)}(t) + \alpha_2 \mathcal{I}_{T(1),T(2)}(t) + \cdots + \alpha_m \mathcal{I}_{T(m-1),T(m)}(t)$, where $0 = T(0) < T(1) < \cdots < T(m) = T$, and the indicator function $\mathcal{I}_{T(i),T(i+1)}(t) = 1$ if $T(i) < t \leq T(i+1)$, and 0 otherwise.

1.2 Global travel model

Our global epidemiological model model is built based on the local model by utilizing travel flow data as follows. For a given country i, suppose the status of its population at the end of day $(t-1)$ is $\mathbf{X}_i(t-1) = [S_i(t-1), I_i(t-1), A_i(t-1), R_i(t-1), D_i(t-1), R_i^u(t-1)]$, and the parameter of the statistical model for this country is $\theta_i = (\alpha_i, \beta_i, \delta_i, \gamma_i)$. On day t, the epidemic state in country i is updated via two steps. First, the state evolves based on country i's internal population. Second, the state evolves based on external factors, here the inflow of airline travelers from other countries and the outflow of airline travelers to other countries.

We consider changes due to internal effects first. For country i, the transition from $t-1$ to t is characterized by the shift from $\mathbf{X}_i(t-1)$ to $\mathbf{X}_i(t) = [S_i(t), I_i(t), A_i(t), R_i(t), D_i(t), R_i^u(t)]$ where

$$\begin{aligned}
S_i(t) &= S_i(t-1) - Y_{1,i}(t-1), & R_i(t) &= R_i(t-1) + Y_{3,i}(t-1),\\
I_i(t) &= I_i(t-1) + Y_{1,i}(t-1) - Y_{2,i}(t-1) - Y_{5,i}(t-1), & D_i(t) &= D_i(t-1) + Y_{4,i}(t-1),\\
A_i(t) &= A_i(t-1) + Y_{2,i}(t-1) - Y_{3,i}(t-1) - Y_{4,i}(t-1), & R_i^u(t) &= R_i^u(t-1) + Y_{5,i}(t-1).
\end{aligned}$$

medRxiv preprint doi: https://doi.org/10.1101/2021.04.14.21255465; this version posted April 20, 2021. The copyright holder for this preprint (which was not certified by peer review) is the author/funder, who has granted medRxiv a license to display the preprint in perpetuity.
It is made available under a CC-BY 4.0 International license .

and $Y_{j,i}(t-1)$, $j = 1, \cdots, 5$, are Poisson distributed with rates

$$h_{1,i}(\mathbf{X}_i(t-1)) = \alpha_i \frac{S_i(t-1)I_i(t-1)}{P_i(t-1)}, \qquad h_2(\mathbf{X}_i(t-1)) = \gamma_i I_i(t-1),$$
$$h_3(\mathbf{X}_i(t-1)) = \beta_i A_i(t-1), \qquad h_4(\mathbf{X}_i(t-1)) = \delta_i A_i(t-1),$$
$$h_5(\mathbf{X}_i(t-1)) = \beta_i I_i(t-1),$$

and $P_i(t-1)$ is the size of the population in country i on day $(t-1)$.

The travel data specify how many new individuals enter the country on day t from each of the disease states. The current state is updated as $\mathbf{X}_i^+(t) = [S_i^+(t), I_i^+(t), A_i^+(t), R_i^+(t), D_i^+(t), R_i^{u+}(t)]$, where $\mathbf{X}_i^+(t) = \mathbf{X}_i(t) + \mathbf{f}_i^{\text{in}}(t) - \mathbf{f}_i^{\text{out}}(t)$, where $\mathbf{f}_i^{\text{in}}(t)$ represents the six compartments of people entering the country on day t and $\mathbf{f}_i^{\text{out}}(t)$ represents the six compartments of people leaving the country on day t. Due to temperature checks and other approaches for screening travelers, we assume that all active confirmed cases are unable to travel. We also assume that deceased individuals do not travel between countries. Consequently, the compartments in $\mathbf{f}_i^{\text{in}}(t)$ and $\mathbf{f}_i^{\text{out}}(t)$ only include four of the six disease states: susceptible (S), undetected infected (I), recovered confirmed (R), and recovered unconfirmed (R^u). Travelers in the recovered confirmed (R) and recovered unconfirmed (R^u) states do not impact the epidemiological state of destination population. However, data on all four categories is not readily available. While each country keeps track of the total number of confirmed recovered each day, they do not necessarily keep track of how many of them leave the country. Therefore, we take a conservative approach and assume that each traveler either belongs to the S category or the I category, meaning travelers bring some potential risk when they arrive in a new country as undetected infected will likely spread the disease and susceptible individuals reduce population immunity and can proliferate disease spread. In other words, we impose $\mathbf{f}_i^{\text{in}}(t) = [S_i^{\text{in}}(t), I_i^{\text{in}}(t), 0, 0, 0, 0]$ and $\mathbf{f}_i^{\text{out}}(t) = [S_i^{\text{out}}(t), I_i^{\text{out}}(t), 0, 0, 0, 0]$, where $I_i^{\text{in}}(t)$ and $I_i^{\text{out}}(t)$ are the number of undetected infected that enter and leave country

medRxiv preprint doi: https://doi.org/10.1101/2021.04.14.21255465; this version posted April 20, 2021. The copyright holder for this preprint
(which was not certified by peer review) is the author/funder, who has granted medRxiv a license to display the preprint in perpetuity.
It is made available under a CC-BY 4.0 International license .

i on day t, respectively. $S_i^{\text{in}}(t)$ and $S_i^{\text{out}}(t)$ are the total numbers of susceptible individuals who enter and leave the country i on day t, respectively. $S_i^{\text{in}}(t) + I_i^{\text{in}}(t) = \text{T}_i^{\text{in}}(t)$ gives the total number of travelers that enter country i on day t, and $S_i^{\text{out}}(t) + I_i^{\text{out}}(t) = \text{T}_i^{\text{out}}(t)$ gives the total number of individuals who leave the country i on day t. As such, $\mathbf{X}_i(t)$ and $\mathbf{X}_i^+(t)$ only differ in the first two categories, where $\mathbf{X}_i^+(t) = [S_i^+(t), I_i^+(t), A_i(t), R_i(t), D_i(t), R_i^u(t)]$, $S_i^+(t) = S_i(t) + S_i^{\text{in}}(t) - S_i^{\text{out}}(t)$, and $I_i^+(t) = I_i(t) + I_i^{\text{in}}(t) - I_i^{\text{out}}(t)$. On day $(t+1)$, the internal model will be updated based on $\mathbf{X}_i^+(t)$. The compartmental quantities are updated as follows

$$\begin{aligned} S_i(t+1) &= S_i^+(t) - Y_{1,i}(t), & R_i(t+1) &= R_i(t) + Y_{3,i}(t), \\ I_i(t+1) &= I_i^+(t) + Y_{1,i}(t) - Y_{2,i}(t) - Y_{5,i}(t), & D_i(t+1) &= D_i(t) + Y_{4,i}(t), \\ A_i(t+1) &= A_i(t) + Y_{2,i}(t) - Y_{3,i}(t) - Y_{4,i}(t), & R_i^u(t+1) &= R_i^u(t) + Y_{5,i}(t). \end{aligned}$$

and $Y_{j,i}(t)$, $j = 1, \cdots, 5$, are Poisson distributed with rates

$$h_{1,i}(\mathbf{X}_i(t)) = \alpha_i \frac{S_i^+(t) I_i^+(t)}{P_i(t)}, \quad h_2(\mathbf{X}_i(t)) = \gamma_i I_i^+(t), \quad h_3(\mathbf{X}_i(t-1)) = \beta_i A_i(t),$$
$$h_4(\mathbf{X}_i(t)) = \delta_i A_i(t), \quad h_5(\mathbf{X}_i(t)) = \beta_i I_i^+(t).$$

Our model assumes that active confirmed cases do not spread the disease due to self-isolation or hospitalization. Therefore, undetected infected cases are the only ones to spread the disease. Moreover, when $I = 0$, the pandemic in the country will cease if we stop admitting undetected infected cases from other countries. Each day, among the people that travel from country i to other countries, there may be some undetected infected cases. If an undetected infected individual enters a country with zero undetected infectious cases, they will seed a new outbreak in this country. Suppose that on day t, there are $I_i^{\text{out}}(t)$ undetected infected people departing from country i to country j, $j \neq i$. Then $I_i^{\text{out}}(t) = \sum_{j=1, j\neq i}^{n} I_{ij}^{\text{out}}(t)$, where $I_{ij}^{\text{out}}(t)$ is the number of undetected infected moving from country i to country j at day t, and n is the total number of

medRxiv preprint doi: https://doi.org/10.1101/2021.04.14.21255465; this version posted April 20, 2021. The copyright holder for this preprint (which was not certified by peer review) is the author/funder, who has granted medRxiv a license to display the preprint in perpetuity.
It is made available under a CC-BY 4.0 International license .

countries. We model the number of undetected infected people who are leaving country i for country j at day t using a multinomial distribution with probabilities based on travel network data. In other words, $\{I_{ij}^{\text{out}}(t)\}_{1\leq j\neq i\leq n} \sim M(I_i^{out}(t), \{p_{ij}(t)\}_{1\leq j\neq i\leq n})$, where $p_{ij}(t) = \frac{\text{T}_{ij}^{\text{out}}(t)}{\text{T}_{i}^{\text{out}}(t)}$ and $\text{T}_{ij}^{\text{out}}(t)$ is the total number of travelers leaving country i for j at day t. Let us denote $S_{ij}^{\text{out}}(t)$ as the number of susceptible people who travel from country i to country j at day t. Then $\text{T}_{ij}^{\text{out}}(t) = S_{ij}^{\text{out}}(t) + I_{ij}^{\text{out}}(t)$. Therefore, at the end of day t, the six states for country i are updated as $\mathbf{X}_i^+(t) = [S_i^+(t), I_i^+(t), A_i(t), R_i(t), D_i(t), R_i^u(t)]$, where

$$S_i^+(t) = S_i(t) + S_i^{\text{in}}(t) - S_i^{\text{out}}(t) = S_i(t) + \sum_{1\leq j\neq i\leq n} S_{ji}^{\text{out}}(t) - S_i^{\text{out}}(t)$$
$$I_i^+(t) = I_i(t) + I_i^{\text{in}}(t) - I_i^{\text{out}}(t) = I_i(t) + \sum_{1\leq j\neq i\leq n} I_{ji}^{\text{out}}(t) - I_i^{\text{out}}(t).$$

1.3 Travel regulation policies

Our goal is to find the value p so that the number of undetected infected $I^+(1), I^+(2), \cdots, I^+(T)$ stay below a given threshold c. More specifically, we consider two types of regulation, an average control policy and a probability control policy described below:

1. Regulation in terms of **average control**, where we find a proportion p such that the average number of undetected cases each day in the next T days stays below a threshold c, i.e., $E(I^+(1)), E(I^+(2)), \cdots, E(I^+(T)) < c$, where E denotes the expectation.

2. Regulation in terms of **probability control**, where we find a proportion p such that the probability of undetected cases each day in the next T days being lower than a threshold c is at least at π, i.e. $P(I^+(1) < c, I^+(2) < c, \cdots, I^+(T)^+ < c) \geq \pi$.

The following lemmas gives us the proportion p that satisfies the above requirements.

Lemma 1. *Under the assumptions of our model, for a given country with population size P, the*

medRxiv preprint doi: https://doi.org/10.1101/2021.04.14.21255465; this version posted April 20, 2021. The copyright holder for this preprint (which was not certified by peer review) is the author/funder, who has granted medRxiv a license to display the preprint in perpetuity.
It is made available under a CC-BY 4.0 International license .

initial status $\mathbf{X}(0) = [S(0), I(0), A(0), R(0), D(0), R^u(0)]$ *and the parameter of the statistical model is* $\theta = (\alpha, \beta, \delta, \gamma)$*, let us denote* $\psi = 1 + \alpha\frac{S(0)}{P} - \gamma - \beta$*,*

1. *The average control requirement is satisfied if*

$$p = \min\left(\frac{(c/I(0))^{1/k}}{\psi} - 1\right)_{k=1,\cdots,T} \tag{1}$$

2. *The probability control requirement is satisfied if*

$$p = \min\left(\frac{(c(1-\pi)/I(0))^{1/k}}{\psi} - 1\right)_{k=1,\cdots,T} \tag{2}$$

Proof:

1. *Average control. With an initial number* $I(0)$ *of undetected infected cases, on the first day, from the internal evolution process we have* $I(1) = I(0) + Y_{1,0} - Y_{2,0} - Y_{5,0}$*, then at the end of this day, we have* $I^+(1) = I(1)(1+p)$*, where* $Y_{1,0} \sim$ *Poisson*$(\alpha\frac{S(0)I(0)}{P})$*,* $Y_{2,0} \sim$ *Poisson*$(\gamma I(0))$ *and* $Y_{5,0} \sim$ *Poisson*$(\beta I(0))$*. Therefore, we have:* $E(I^+(1) = E\big(E(I^+(1)/I(0))\big) = I(0)(1 + \alpha\frac{S(0)}{P} - \gamma - \beta)(1+p) < I(0)\psi(1+p)$*. Similarly, we have:* $E(I^+(2)) = E\big(E(I^+(2)\ /I^+(1))\big) = E\big(I^+(1)(1+\alpha\frac{S(1)}{P} - \gamma - \beta)(1+p)\big) < (1+p)\psi E(I_1^+) = (1+p)^2\psi^2 I(0)$*. Repeating this argument until reaching day* T *results in* $E(I(T)^+) < (1+p)^T\psi^T I_0$*. For average control, therefore, we want to find a* p *such that:* $(1+p)\psi I(0), (1+p)^2\psi^2 I(0), \cdots, (1+p)^T\psi^T I(0) < c$*. By solving the above inequality, the* p *that satisfies the requirements is*

$$p = \min\left(\frac{(c/I(0))^{1/k}}{\psi} - 1\right)_{k=1,\cdots,T} \blacksquare$$

medRxiv preprint doi: https://doi.org/10.1101/2021.04.14.21255465; this version posted April 20, 2021. The copyright holder for this preprint
(which was not certified by peer review) is the author/funder, who has granted medRxiv a license to display the preprint in perpetuity.
It is made available under a CC-BY 4.0 International license .

2. *Probability control. Here we have*

$$E\big(I^+(1)/I(0)\big) = \psi(1+p)I(0), E\big(I^+(2)/I^+(1)\big) < \psi(1+p)I^+(1),$$
$$E\big(I^+(3)/I^+(2)\big) < \psi(1+p)I^+(2), \cdots, E\big(I^+(T)/I^+(T-1)\big) < \psi(1+p)I^+(T-1).$$

Let us denote $\frac{I^+(1)}{\psi(1+p)} = I^*(1), \frac{I^+(2)}{\psi^2(1+p)^2} = I^*(2), \cdots, \frac{I^+(T)}{\psi^T(1+p)^T} = I^*(T)$. *The sequence* $I^*(1), I^*(2), \cdots, I^*(T)$ *forms a non-negative supermartingale sequence. Since*

$$E\big(I^*(1)/I(0)\big) = I(0), E\big(I^*(2)/I^*(1)\big) < I^*(1),$$
$$E\big(I^*(3)/I^*(2)\big) < I^*(2), \cdots, E\big(I^*(T)/I^*(T-1)\big) < I^*(T-1).$$

Applying the maximal inequality for a non-negative supermartingale we have: $P(\cup_{i\geq 1}(I^*(i) \geq m)) \leq \frac{E(I^*(1))}{m}$*, for a given* $m > 0$. *This gives* $1 - P(\cup_{i\geq 1}(I^*(i) \geq m)) \geq 1 - \frac{E(I^*(1))}{m}$. *In other words,* $P(I^*(1) < m, I^*(2) < m, \cdots, I^*(T) < m) \geq 1 - \frac{E(I^*(1))}{m} = 1 - \frac{I(0)}{m}$. *If we want* $P(I^*(1) < m, I^*(2) < m, \cdots, I^*(T) < m) \geq p_c$*, then the smallest value of* m *must satisfy the relation* $1 - \frac{I(0)}{m} = \pi$. *We choose* $m = \frac{I(0)}{1-\pi}$. *We have:* $P(I^*(1) < m, I^*(2) < m, \cdots, I^*(T) < m) = P\big(I^+(1) < m\psi(1+p), I^+(2) < m\psi^2(1+p)^2, \cdots, I^+(T) < m\psi^T(1+p)^T\big)$. *So, if we want* $I^+(1), I^+(2), \cdots, I^+(T) < c$*, then we need to find a p such that* $m\psi(1+p) < c, m\psi^2(1+p) < c, \cdots, m\psi^T(1+p)^T < c$. *In other words, we need a* p *that satisfies:* $p < \frac{(c/m)^{1/k}}{\psi} - 1, \forall k = 1, \cdots, T$, *or* $p < \frac{(c(1-\pi)/I(0))^{1/k}}{\psi} - 1, \forall k = 1, \cdots, T$. *In conclusion, for a probability control level* π *and a threshold* c *in the next* T *days, the required* p *is*

$$p = \min\left(\frac{(c(1-\pi)/I(0))^{1/k}}{\psi} - 1\right)_{k=1,\cdots,T} \blacksquare$$

Remark: The probability control policy is more conservative than the average control policy. Under the same threshold c*, the difference between the two policies is the factor* $(1-\pi)$ *in the*

medRxiv preprint doi: https://doi.org/10.1101/2021.04.14.21255465; this version posted April 20, 2021. The copyright holder for this preprint
(which was not certified by peer review) is the author/funder, who has granted medRxiv a license to display the preprint in perpetuity.
It is made available under a CC-BY 4.0 International license .

numerator of the probability control strategy. If we want the probability control to have at least 0.9 of the threshold c of average control, this factor becomes 0.1. As a result, the proportion of p in probability control is much smaller than the proportion of p in average control. If we want to use the probability control with a probability of at least 0.9, we need to set up the threshold c in probability control higher than the threshold c in average control to make sure that our p is non-negative.

Example 1. Here we give one example of using the average control policy to regulate the travel. For simplicity, we consider a small world with only three countries, with the following initial states and true parameter values:

$$\mathbf{X}_1(0) = [S_1(0), I_1(0), A_1(0), R_1(0), D_1(0), R_1^u(0)] = (28718795, 68, 167, 259, 149, 101)$$

$$\theta_1 = (\alpha_1, \beta_1, \delta_1, \gamma_1) = (0.82, 0.18, 0.09, 0.68)$$

$$\mathbf{X}_2(0) = (6358016, 40356, 1573, 454, 55, 320)$$

$$\theta_2 = (0.74, 0.15, 0.02, 0.06)$$

$$\mathbf{X}_3(0) = (28507087, 206, 764, 619, 72, 188)$$

$$\theta_3 = (0.92, 0.13, 0.02, 0.76)$$

and we want to regulate the incoming travel in the first country (country 1). These choices of initial conditions and parameter values are based on our simulations where benefits of travel restriction can be seen clearly. We now need to find the regulation sequences $\{p_{21}(t)\}_{t=1,\cdots,7}$ and $\{p_{31}(t)\}_{t=1,\cdots,7}$ that can regulate airline travel from country 2 to country 1 and from country 3 country 1 such that for the next $T = 7$ days, the number of undetected infected cases in the arriving country will not exceed $c = 70$ cases on average per day. Applying Lemma 1a, we can find parameter p for country 1 as $p = \min\left((c/I_0)^{1/k}/\psi - 1\right)_{k=1,\cdots,7} = 0.035$.

So the sequence of the number of undetected infected imported cases that country 1 can

medRxiv preprint doi: https://doi.org/10.1101/2021.04.14.21255465; this version posted April 20, 2021. The copyright holder for this preprint (which was not certified by peer review) is the author/funder, who has granted medRxiv a license to display the preprint in perpetuity.
It is made available under a CC-BY 4.0 International license .

accept each day as $\left(\left((1+p)\psi\right)^{i} I(0)\right)_{i=1,\cdots,7} = (2,2,2,2,2,2,2)$. Because country 1 has two "neighbors", so country 1 can accept about 1 undetected infected from each neighbor each day during the regulation period.

The next step is to predict, for each day, the number of undetected infected travelers from countries 2 and 3 that can enter country 1 for the next 7 days when full travel is allowed. We get these numbers by simulating data given the true parameters under the fully open scenario. We first simulate 10000 stochastic realizations under this scenario and use the 0.975 percentile of the simulated sequence of undetected infected in countries 2 and 3 in the next 7 days as proxies for the number of undetected infected cases in these countries. Then we simulate a deterministic realization under the fully open scenario during the regulation period and use the values from the deterministic realization to calculate the percentage of undetected infected people in countries 2 and 3. Based on these percentages and the travel data, we estimate how many undetected infected individuals enter country 1 from country 2 and country 3 daily during the regulation period if full travel is allowed. The final step is obtaining the regulation sequence that country 1 can allow country 2 and country 3 to enter its border. The regulation sequence that country 1 can allow for country 2 to enter its border during the regulation period is obtained by dividing the number of daily undetected infected cases that country 1 can tolerate from country 2 by the daily estimated number of imported undetected infected cases from country 2 if full travel is allowed. Notice that if the daily proportion is greater or equal to 1, we set it to 1. Repeat the same procedure, we can also find the regulation sequence that country 1 can allow for country 3 to enter its border.

Following the above steps, we can find that in the next 7 days, the regulation sequence of proportions of people who can move from country 2 to country 1 is (0.103, 0.076, 0.051, 0.035, 0.022, 0.014, 0.009), and the sequence of proportions of people who can move from country 3 to country 1 is (1, 1, 1, 1, 1, 1, 1). Compared to the fully open scenario, using the average control approach with the threshold of 70 cases during the 7-day regulation period, about 6.03%

medRxiv preprint doi: https://doi.org/10.1101/2021.04.14.21255465; this version posted April 20, 2021. The copyright holder for this preprint (which was not certified by peer review) is the author/funder, who has granted medRxiv a license to display the preprint in perpetuity.
It is made available under a CC-BY 4.0 International license .

of travelers from country 2 are allowed enter the country 1, and all travelers from country 3 are allowed to enter country 1. Overall, the volume of inbound travelers in country 1 is about 88.64% of the normal levels.

In practice, the value of the transmission rate α varies over time, and we therefore provide an additional lemma that generalizes Lemma 1 to address the aspect of varying α.

Lemma 2. *Under the assumptions of our model, for a given country with population size* P*, initial status* $\mathbf{X}(0) = [S(0), I(0), A(0), R(0), D(0), R^u(0)]$ *and the parameter of the statistical model for this country over the time period from* $0 = T(0)$ *to* $T = T(m)$ *is* $\theta = (\alpha, \beta, \delta, \gamma)$*, where* $\alpha = \alpha_1 \mathcal{I}_{0,T(1)}(t) + \alpha_2 \mathcal{I}_{T(1),T(2)}(t) + \cdots + \alpha_m \mathcal{I}_{T(m-1),T(m)}(t)$*. Let us denote* $\psi_{max} = max\left(1 + \alpha_i \frac{S(0)}{P} - \gamma - \beta\right)_{i=1,\ldots,m}$.

1. *The average control requirement is satisfied if*

$$p = \min\left(\frac{(c/I_0)^{1/k}}{\psi_{max}} - 1\right)_{k=1,\cdots,T} \tag{3}$$

2. *The probability control requirement is satisfied if*

$$p = \min\left(\frac{(c(1-\pi)/I_0)^{1/k}}{\psi_{max}} - 1\right)_{k=1,\cdots,T} \tag{4}$$

Proof:

1. *Average control. Follow the same argument as in the proof of Lemma 1, we have* $E(I^+(1) = E\big(E(I^+(1)/I(0))\big) = I(0)(1 + \alpha\frac{S(0)}{P} - \gamma - \beta)(1+p) = I(0)(1 + \alpha_1\frac{S(0)}{P} - \gamma - \beta)(1+p) < I(0)\psi_{max}(1+p)$*. Repeating the argument until we reach day* T *yields :* $E(I(T)^+) < (1+p)^T \psi_{max}^T I(0)$*. Therefore, we want to find a* p *such that* $(1+p)\psi_{max}I(0), (1+p)^2\psi_{max}^2 I(0), \cdots, (1+p)^T\psi_{max}^T I(0) < c$*. Hence the value* p *that*

medRxiv preprint doi: https://doi.org/10.1101/2021.04.14.21255465; this version posted April 20, 2021. The copyright holder for this preprint (which was not certified by peer review) is the author/funder, who has granted medRxiv a license to display the preprint in perpetuity.
It is made available under a CC-BY 4.0 International license .

satisfies the requirements for average control is

$$p = \min\left(\frac{(c/I(0))^{1/k}}{\psi_{max}} - 1\right)_{k=1,\cdots,T} \blacksquare$$

2. *Probability control. Here we have*

$$E\big(I^+(1)/I(0)\big) < \psi_{max}(1+p)I(0), E\big(I^+(2)/I^+(1)\big) < \psi_{max}(1+p)I^+(1),$$
$$E\big(I^+(3)/I^+(2)\big) < \psi_{max}(1+p)I^+(2), \cdots, E\big(I^+(T)/I^+(T-1)\big) < \psi_{max}(1+p)I^+(T-1).$$

Let us denote $\frac{I^+(1)}{\psi_{max}(1+p)} = I^*(1), \frac{I^+(2)}{\psi_{max}^2(1+p)^2} = I^*(2), \cdots, \frac{I^+(T)}{\psi_{max}^T(1+p)^T} = I^*(T)$. *Similar to Lemma 1, the sequence of* $I^*(1), I^*(2), \cdots, I^*(T)$ *forms a non-negative supermartingale sequence. Hence, following the same arguments as in Lemma 1, we have for a probability control level* π *and a threshold* c *in the next* T *days, the required* p *is*

$$p = \min\left(\frac{(c(1-\pi)/I(0))^{1/k}}{\psi_{max}} - 1\right)_{k=1,\cdots,T} \blacksquare$$

1.4 Choosing distance and summary statistics in Approximation Bayesian Computational

There are many variants of ABC, but they are all based on a comparison of observed and simulated data, which in most cases requires specification of data summary statistics, a distance measure, and a scalar distance threshold ϵ. The most basic ABC algorithm, the so-called accept-reject method, starts by simulating a parameter value from a prior distribution and then uses the model, given this parameter value, to generate one realization of data. If the distance between the summary statistics for the observed data and the summary statistics for the simulated data is less than or equal to ϵ, the sampled parameter value is retained; otherwise, it is discarded. The collection of accepted parameter values constitutes a sample from an approximation of the

medRxiv preprint doi: https://doi.org/10.1101/2021.04.14.21255465; this version posted April 20, 2021. The copyright holder for this preprint (which was not certified by peer review) is the author/funder, who has granted medRxiv a license to display the preprint in perpetuity.
It is made available under a CC-BY 4.0 International license .

posterior distribution. The approximation generally improves with smaller values of ϵ, but at the same time it becomes more computationally expensive to obtain acceptances.

This basic ABC algorithm is computationally inefficient when working with a small threshold ϵ as a vast majority of sampled parameter values are rejected. To address this inefficiency, some sequential variants of ABC have been proposed, such as the ABC Markov chain Monte Carlo algorithm (ABC-MCMC) by Marjoram, et al. (2003) and the ABC Sequential Monte Carlo algorithm (ABC-SMC) by Sisson et al. (2007), Toni, et al. (2009), and Drovandi and Pettitt (2011) (*3–6*). In this paper, we use the variant from Drovandi and Pettitt (2011) (*6*), called replenishment ABC (RABC). For its implementation, we use the R package protoABC from Ebert (2020) (*7*). This package is very flexible as the users can employ any priors, generative models, and distance functions.

A commonly used distance is the Euclidean distance due to its simple form. In our problem setting, this Euclidean distance $L\left(S(\text{Data}^{(i)}), S(\text{Data})\right)$ can be written as:

$$\left(\frac{1}{T}\sum_{t=1}^{T}\left[(A^{(i)}(t)-A(t))^2+(R^{(i)}(t)-R(t))^2+(D^{(i)}(t)-D(t))^2\right]\right)^{1/2},$$

where $\left(A^{(i)}(t), R^{(i)}(t), D^{(i)}(t)\right)$ and $\left(A(t), R(t), D(t)\right)$ are active confirmed, accumulated recovered confirmed, and accumulated death confirmed cases on day t of the simulated data and the real (empirical) data, respectively. However, simply using the Euclidean distance may not be the best choice since it does not account for the scale of different quantities, and may need to be standardized (see for example Beaumont et al. (2002), Csilléry et al. (2012), or Prangle (2017) (*8–10*)). This is why we also consider the following weighted Euclidean distance, with weights given by the inverse variances:

$$\left(\frac{1}{T}\sum_{t=1}^{T}\left[\left(\frac{A^{(i)}(t)-A(t)}{\text{sd}(A(t))}\right)^2+\left(\frac{R^{(i)}(t)-R(t)}{\text{sd}(R(t))}\right)^2+\left(\frac{D(t)^{(i)}-D(t)}{\text{sd}(D(t))}\right)^2\right]\right)^{1/2},$$

medRxiv preprint doi: https://doi.org/10.1101/2021.04.14.21255465; this version posted April 20, 2021. The copyright holder for this preprint (which was not certified by peer review) is the author/funder, who has granted medRxiv a license to display the preprint in perpetuity.
It is made available under a CC-BY 4.0 International license .

where $\{\text{sd}(A(t)),\ \text{sd}(R(t)),\ \text{sd}(D(t))\}_{t=1,\cdots,T}$ are the prior predictive standard deviations of $A(t), R(t), D(t)$ at each time step. These standardization values are obtained by first generating N (N being large) parameters values $\theta = (\alpha, \beta, \delta, \gamma)$ from the prior $\pi(\theta)$, and then generating one realization of simulated data for each of them. The standard deviation at each time step is then calculated based on these N simulated data, giving $\{\text{sd}\,(A(t)),\ \text{sd}\,(R(t)),\ \text{sd}\,(D(t))\}_{t=1,\cdots,T}$. In our simulation study, we choose $N = 5000$.

To improve the predictive quality of ABC algorithms, we can also use additional information from parameter estimates to make new summary statistics. Under our model assumptions, we can learn additional knowledge about how parameters can be estimated. We are thus going to include as summary statistics estimates of our epidemiological parameters. For simplicity, we first limit our discussion to the local model, where each country is considered separately. The choice of the distance for the global model will be discussed in Section 1.5.

Under our model assumptions, we have: $R(t) = R(t-1) + \text{Poisson}\,(\beta A(t-1))\,, \forall t = 1, \cdots, T$. So for a given $A(t-1)$, we have: $E\,(R(t) - R(t-1)) = \beta A(t-1)$. This yields $E\left(\frac{R(t)-R(t-1)}{A(t-1)}\right) = \beta$. So for a given sequence of known $\{A(t)\}_{t=1,\cdots,T}$, the sequence of independent variables $\{\frac{R(t)-R(t-1)}{A(t-1)}\}_{t=1,\cdots,T}$ has β as common mean value. Therefore, we can use its median value to estimate β. If our algorithm generated a reasonable $\theta^{(i)}$, then the data generated by $\theta^{(i)}$ should also gives us a sequence $\{\frac{R^{(i)}(t)-R^{(i)}(t-1)}{A^{(i)}(t-1)}\}_{t=1,\cdots,T}$ with median value close to the corresponding median value of $\{\frac{R(t)-R(t-1)}{A(t-1)}\}_{t=1,\cdots,T}$. Therefore under our model assumptions, adding the term $|\text{median}\{\frac{R(t)-R(t-1)}{A(t-1)}\}_{t=1,\cdots,T} - \text{median}\{\frac{R^{(i)}(t)-R^{(i)}(t-1)}{A^{(i)}(t-1)}\}_{t=1,\cdots,T}|$ when calculating $L\big(S(\text{Data}^{(i)}), S(\text{Data})\big)$ would help to improve the estimation of β. Similarly, the median of the sequence $\{\frac{D(t)-D(t-1)}{A(t-1)}\}_{t=1,\cdots,T}$ can be used to estimate the death rate δ, and adding the term $|\text{median}\{\frac{D(t)-D(t-1)}{A(t-1)}\}_{t=1,\cdots,T} - \text{median}\{\frac{D^{(i)}(t)-D^{(i)}(t-1)}{A^{(i)}(t-1)}\}_{t=1,\cdots,T}|$ would help to improve the estimation of δ.

We now try to learn the transmission rate α under our model assumptions. We have $S(t) =$

medRxiv preprint doi: https://doi.org/10.1101/2021.04.14.21255465; this version posted April 20, 2021. The copyright holder for this preprint (which was not certified by peer review) is the author/funder, who has granted medRxiv a license to display the preprint in perpetuity.
It is made available under a CC-BY 4.0 International license .

$S(t-1)+\text{Poisson}\left(\alpha\frac{S(t-1)I(t-1)}{P}\right), \forall t=1,\cdots,T$, where P is the total population size of the country. So for a given $S(t-1), I(t-1)$, we have $E\left(S(t)-S(t-1)\right)=\alpha\frac{S(t-1)I(t-1)}{P}$. This yields $E\left(\frac{S(t)-S(t-1)}{S(t-1)I(t-1)}P\right)=\alpha$. Unfortunately, $S(t-1)$ and $I(t-1)$ are hidden states and not available in our data. To use the above strategy to improve the estimation of α, we need to reconstruct these hidden states based on the available data $\{(A(t),R(t),D(t))\}_{t=1,\cdots,T}$. Because our model is stochastic, all values would change each time we rerun the model. However, based on the available data $\{(A(t),R(t),D(t))\}_{t=1,\cdots,T}$ we can reconstruct the mean realization that adopts these three states. Let us denote $U(t)$ the total number of confirmed cases at time t, $\Delta U(t-1)$ the number of new confirmed cases at time t as, and $\Delta R^u(t-1)$ the number of new undocumented recover cases at day t. Note that $U(t)=A(t)+R(t)+D(t)$, $\Delta U(t-1)=U(t)-U(t-1)$, and $\Delta R^u(t-1)=R^u(t)-R^u(t-1)$.

From the local model we have:

$$\begin{aligned}U(t)&=A(t)+R(t)+D(t)\\&=A(t-1)+Y_2(t-1)-Y_3(t-1)-Y_4(t-1)\\&\qquad+R(t-1)+Y_3(t-1)+D(t-1)+Y_4(t-1)\\&=A(t-1)+R(t-1)+D(t-1)+Y_2(t-1)\\&=U(t-1)+Y_2(t-1)\end{aligned}$$

So $Y_2(t-1)=U(t)-U(t-1)=\Delta U(t-1)$. Moreover, since $Y_2(t-1)\sim\text{Poisson}\left(\gamma I(t-1)\right)$, we have

$$E\left(I(t-1)\right)=E\left(\frac{\Delta U(t-1)}{\gamma}\right). \tag{5}$$

Equation (5) tells us that with the observed data $\{A(t),R(t),D(t)\}_{t=1,\cdots,T}$, if the identification rate γ is known, the average realization $I(t)$ can be reconstructed up to time $T-1$.

Similarly, since $R^u(t)=R^u(t-1)+Y_5(t-1)$, where $Y_5(t-1)\sim\text{Poisson}\left(\beta I(t-1)\right)$, we

medRxiv preprint doi: https://doi.org/10.1101/2021.04.14.21255465; this version posted April 20, 2021. The copyright holder for this preprint (which was not certified by peer review) is the author/funder, who has granted medRxiv a license to display the preprint in perpetuity.
It is made available under a CC-BY 4.0 International license .

have

$$E\left(\Delta R^u(t-1)\right) = E\left(\beta I(t-1)\right).$$

Using (5), we obtain

$$E\left(\Delta R^u(t-1)\right) = E\left(\frac{\beta \Delta U(t-1)}{\gamma}\right). \quad (6)$$

Moreover, using $R^u(t) = R^u(0) + \sum_{i=1}^{t-1} \Delta R^u(i)$ and (6), the average value of $R^u(t)$ can be reconstructed as

$$\begin{aligned} E\left(R^u(t)\right) &= E\left(R^u(0)\right) + \sum_{i=1}^{t-1} E\left(\Delta R^u(i)\right) \\ &= E\left(R^u(0)\right) + \sum_{i=1}^{t-1} E\left(\beta I(i)\right) \\ &= E\left(R^u(0)\right) + \sum_{i=1}^{t-1} E\left(\frac{\beta \Delta U(i)}{\gamma}\right) \end{aligned} \quad (7)$$

Equations (5) and (7) tell us that based on the available data $\{(A(t), R(t), D(t)\}_{t=1,\cdots,T}$ if the identification rate γ and the recovered rate β are available to us, then we can reconstruct the average realization of $I(t)$ and $R^u(t), \forall t = 1, \cdots, T-1$. As a result the average realization category at time t can also be reconstruct as $P - E\left(I(t) - A(t) - R(t) - D(t) - R^u(t)\right) = P - U(t) - \frac{\Delta U(t)}{\gamma} - \sum_{i=0}^{t-1} \frac{\beta}{\gamma} \Delta U(i)$, where P is the country population.

Overall, based on the observed data $\{(A(t), R(t), D(t))\}_{t=1,\cdots,T}$, suppose that the identification rate γ and the recover rate β are available to us. The average realization that adopts

medRxiv preprint doi: https://doi.org/10.1101/2021.04.14.21255465; this version posted April 20, 2021. The copyright holder for this preprint (which was not certified by peer review) is the author/funder, who has granted medRxiv a license to display the preprint in perpetuity.
It is made available under a CC-BY 4.0 International license .

$\{(A(t), R(t), D(t))\}_{t=1,\cdots,T}$ can be reconstructed up to time $T-1$ as

$$\begin{aligned}&\{S(t), I(t), A(t), R(t), D(t), R^u(t)\}_{t=1,\cdots,T-1}\\=&\{P(t)-U(t)-\frac{\Delta U(t)}{\gamma}-\sum_{i=0}^{t-1}\frac{\beta}{\gamma}\Delta U(i), \frac{\Delta U(t)}{\gamma}, A(t), R(t), D(t), \sum_{i=0}^{t-1}\frac{\beta}{\gamma}\Delta U(i)\}_{t=1,\cdots,T-1}.\end{aligned}$$

Therefore α can be estimated as the median of the sequence $\{\frac{S(t-1)-S(t)}{S(t-1)I(t-1)}P\}_{1,\cdots,T-1}$. Unfortunately, β and γ are not known in advance and need to be estimated.

We therefore use the testing argument to recover the average realization and estimate α. This argument is based on the following observation. In step 1 of the ABC algorithm, a parameter value $\theta^{(i)} = \left(\alpha^{(i)}, \beta^{(i)}, \delta^{(i)}, \gamma^{(i)}\right)$ is generated and available to us. If $\gamma^{(i)}, \beta^{(i)}$ are correctly specified as γ, β of the underlying true parameter $\theta = (\alpha, \beta, \delta, \gamma)$. We would expect that the median value of the sequence $\{\frac{S(t-1)-S(t)}{S(t-1)I(t-1)}P\}_{1,\cdots,T-1}$ constructed using $\gamma^{(i)}, \beta^{(i)}$ and the available data $\{(A(t), R(t), D(t))\}_{t=1,\cdots,T}$, should give us a good estimator for the underlying true α value. A similar statement holds for the median value of the sequence $\{\frac{S^{(i)}(t-1)-S^{(i)}(t)}{S^{(i)}(t-1)I^{(i)}(t-1)}P\}_{1,\cdots,T-1}$ that was constructed by $\gamma^{(i)}, \beta^{(i)}$ and the available data $\{(A^{(i)}(t), R^{(i)}(t), D^{(i)}(t)\}_{t=1,\cdots,T}$ should also give us a good estimator for the underlying true α value. Therefore the distance $|\text{median}\{\frac{S(t-1)-S(t)}{S(t-1)I(t-1)}P\}_{t=1,\cdots,T-1} - \text{median}\{\frac{S^{(i)}(t-1)-S^{(i)}(t)}{S^{(i)}(t-1)I^{(i)}(t-1)}P\}_{t=1,\cdots,T-1}|$ in $L\left(S(\text{Data}^{(i)}), S(\text{Data})\right)$ should be close to 0. On the other hand, if the generated parameters $\gamma^{(i)}, \beta^{(i)}$ are far away from the underlying true parameters, then $|\text{median}\{\frac{S^{(i)}(t)-S^{(i)}(t-1)}{S^{(i)}(t-1)I^{(i)}(t-1)}P\}_{t=1,\cdots,T-1} - \text{median}\{\frac{S(t)-S(t-1)}{S(t-1)I(t-1)}P\}_{t=1,\cdots,T-1}|$ should not be close to 0.

Based on this observation, we then add the term

$$|\text{median}\{\frac{S^{(i)}(t)-S^{(i)}(t-1)}{S^{(i)}(t-1)I^{(i)}(t-1)}P\}_{t=1,\cdots,T-1} - \text{median}\{\frac{S(t)-S(t-1)}{S(t-1)I(t-1)}P\}_{t=1,\cdots,T-1}|$$

in $L\big(S(\text{Data}^{(i)}), S(\text{Data})\big)$ to improve the estimation for α.

medRxiv preprint doi: https://doi.org/10.1101/2021.04.14.21255465; this version posted April 20, 2021. The copyright holder for this preprint (which was not certified by peer review) is the author/funder, who has granted medRxiv a license to display the preprint in perpetuity.
It is made available under a CC-BY 4.0 International license .

Finally, our proposed distance for the model is designed as follows:

$$\sqrt{\frac{1}{T}\sum_{t=1}^{T}[\left(\frac{A^{(i)}(t)-A(t)}{\text{sd}(A(t))}\right)^2+\left(\frac{R^{(i)}(t)-R(t)}{\text{sd}(R(t))}\right)^2+\left(\frac{D^{(i)}(t)-D(t)}{\text{sd}(D(t))}\right)^2]}+\sqrt{d},$$

where

$$\begin{aligned} d = &|\text{median}\{\frac{R(t)-R(t-1)}{A(t-1)}\}_{t=1,\cdots,T} - \text{median}\{\frac{R^{(i)}(t)-R^{(i)}(t-1)}{A^{(i)}(t-1)}\}_{t=1,\cdots,T}| \\ &+ |\text{median}\{\frac{D(t)-D(t-1)}{A(t-1)}\}_{t=1,\cdots,T} - \text{median}\{\frac{D^{(i)}(t)-D^{(i)}(t-1)}{A^{(i)}(t-1)}\}_{t=1,\cdots,T}| \\ &+ |\text{median}\{\frac{S(t-1)-S(t)}{S(t-1)I(t-1)}P\}_{t=1,\cdots,T-1} - \text{median}\{\frac{S^{(i)}(t-1)-S^{(i)}(t)}{S^{(i)}(t-1)I^{(i)}(t-1)}P\}_{t=1,\cdots,T-1}|. \end{aligned}$$

1.5 Marginal approach to parameter estimation

In the following, we will discuss how to use ABC to estimate parameters in each country for our global model. The challenge of using ABC to estimate the global model parameters is that many parameters need to be estimated. Therefore directly using ABC to estimate all the parameters for all countries at once may result in very unstable parameter estimations and will be extremely computationally intensive. We propose a marginal estimating approach to estimate each country parameter for the global model separately while still taking into account the travel data.

For simplicity, let us first consider a given country m with the represented parameter $\theta_m = (\alpha_m, \beta_m, \delta_m, \gamma_m)$. Let us denote $A_k(t), R_k(t), D_k(t)$ as the number of active confirm cases, accumulated recover confirmed, and accumulated death confirmed at country k on day t, respectively. We denote $T(t) = [T_{ij}(t)]_{i,j=1,\cdots,n}$ is the global traveling matrix at day t, where $T_{ij}(t)$ is the number of travelers from country i to country j at day t. Notice that $T_{ij}(t) = 0$ if $i = j, \forall t \in 1,\cdots,T$. With the available data from the global model $\{A_k(t), R_k(t), D_k(t)\}_{k=1,\cdots,n;t=1,\cdots,T}$ and the travel data $\{T(t)\}_{t=1,\cdots,T}$, we need to estimate θ_i.

Before introducing our estimation procedure, we rewrite how our global model evolved for

medRxiv preprint doi: https://doi.org/10.1101/2021.04.14.21255465; this version posted April 20, 2021. The copyright holder for this preprint (which was not certified by peer review) is the author/funder, who has granted medRxiv a license to display the preprint in perpetuity.
It is made available under a CC-BY 4.0 International license .

the country m at day t and $(t+1)$:

Step 1a *The internal pandemic evolves at day t:* The internal pandemic situation in the country evolves from $\mathbf{X}_m(t-1)$ to $\mathbf{X}_m(t) = [S_m(t), I_m(t), A_m(t), R_m(t), D_m(t), R_m^u(t)]$, where $S_m(t) = S_m(t-1) - Y_{1,m}(t-1)$, $I_m(t) = I_m(t-1) + Y_{1,m}(t-1) - Y_{2,m}(t-1) - Y_{5,m}(t-1)$, $A_m(t) = A_m(t-1) + Y_{2,m}(t-1) - Y_{3,m}(t-1) - Y_{4,m}(t-1)$, $R_m(t) = R_m(t-1) + Y_{3,m}(t-1)$, $D_m(t) = D_m(t-1) + Y_{4,m}(t-1)$, $R_m^u(t) = R_m^u(t-1) + Y_{5,m}(t-1)$. And $Y_{j,m}(t-1), j = 1, \cdots, 5$ are Poisson distributed with rates $(h_{j,i}(\mathbf{X}_m(t-1)))$, respectively, as $h_{1,m}(\mathbf{X}_m(t-1)) = \alpha_m \frac{S_m(t-1)I_i(t-1)}{P_m(t-1)}$, $h_2(\mathbf{X}_m(t-1)) = \gamma_m I_m(t-1)$, $h_3(\mathbf{X}_m(t-1)) = \beta_m A_m(t-1)$, $h_4(\mathbf{X}_m(t-1)) = \delta_m A_m(t-1)$, and $h_5(\mathbf{X}_m(t-1)) = \beta_m I_m(t-1)$.

Step 1b *The external pandemic added at day t:* From the travel data, $\mathbf{X}_m(t)$ is updated to $\mathbf{X}_m^+(t) = [S_m^+(t), I_m^+(t), A_m(t), R_m(t), D_m(t), R_m^u(t)]$, where $S_m^+(t) = S_m(t) + S_m^{\text{in}}(t) - S_m^{\text{out}}(t)$, and $I_m^+(t) = I_m(t) + I_m^{\text{in}}(t) - I_m^{\text{out}}(t)$.

Step 2a *The internal pandemic evolves at day $(t+1)$:* The internal pandemic situation in the country evolves from $\mathbf{X}_m^+(t)$ to $\mathbf{X}_m(t+1) = [S_m(t+1), I_m(t+1), A_m(t+1), R_m(t+1), D_m(t+1), R_m^u(t+1)]$, as $S_m(t+1) = S_m^+(t) - Y_{1,m}(t)$, $I_m(t+1) = I_m^+(t) + Y_{1,m}(t) - Y_{2,m}(t) - Y_{5,m}(t)$, $A_m(t+1) = A_m(t) + Y_{2,m}(t) - Y_{3,m}(t) - Y_{4,m}(t)$, $R_m(t+1) = R_m(t) + Y_{3,m}(t)$, $D_m(t+1) = D_m(t) + Y_{4,m}(t)$, $R_m^u(t+1) = R_m^u(t) + Y_{5,m}(t)$. And $Y_{j,m}(t), j = 1, \cdots, 5$ are Poisson distributed with rates $h_{j,m}(\mathbf{X}_m(t))$, respectively, as $h_{1,m}(\mathbf{X}_m(t)) = \alpha_m \frac{S_m^+(t)I_m^+(t)}{P_m(t)}$, $h_2(\mathbf{X}_m(t)) = \gamma_m I_m^+(t)$, $h_3(\mathbf{X}_m(t-1)) = \beta_i A_i(t)$, $h_4(\mathbf{X}_i(t)) = \delta_i A_i(t)$, and $h_5(\mathbf{X}_m(t)) = \beta_m I_m^+(t)$.

Step 2b *The external pandemic added at day $(t+1)$:* From the travel data, $\mathbf{X}_m(t+1)$ is updated to $\mathbf{X}_m^+(t+1) = [S_m^+(t+1), I_m^+(t+1), A_m(t), R_m(t+1), D_m(t+1), R_m^u(t+1)]$, where $S_m^+(t+1) = S_m(t+1) + S_m^{\text{in}}(t+1) - S_m^{\text{out}}(t+1)$, and $I_m^+(t+1) = I_m(t+1) + I_m^{\text{in}}(t+1) - I_m^{\text{out}}(t)$.

medRxiv preprint doi: https://doi.org/10.1101/2021.04.14.21255465; this version posted April 20, 2021. The copyright holder for this preprint (which was not certified by peer review) is the author/funder, who has granted medRxiv a license to display the preprint in perpetuity.
It is made available under a CC-BY 4.0 International license .

As shown in the model's evolving process, step 1b and step 2b make the global model behave differently from the local model. Therefore, if we can estimate quantities $S_m^{\text{in}}(t)$, $I_m^{\text{in}}(t)$, $S_m^{\text{out}}(t)$, $I_m^{\text{out}}(t)$ for each day t, then we can use the marginal approach to estimate each country's parameters separately. The two quantities $S_m^{\text{out}}(t)$, $I_m^{\text{out}}(t)$ come from inside the considered country m. Therefore the amount can be calculated during the data generation process of the ABC algorithm. Our main task now is estimating $S_m^{\text{in}}(t)$, $I_m^{\text{in}}(t)$.

We have $S_m^{\text{in}}(t) = \sum_{1 \le j \ne m \le n} S_{jm}^{\text{out}}(t)$, $I_m^{\text{in}}(t) = \sum_{1 \le j \ne m \le n} I_{jm}^{\text{out}}(t)$, where $S_{jm}^{\text{out}}(t)$ and $I_{jm}^{\text{out}}(t)$ are the number of susceptible people and undocumented infected people move from country j to country m at day t, respectively. It should be noticed that under our model assumptions the summation of $S_{jm}^{\text{out}}(t)$ and $I_{jm}^{\text{out}}(t)$ gives us the total number of people traveling from country j to country m at day t, i.e. $S_{jm}^{\text{out}}(t) + I_{jm}^{\text{out}}(t) = T_{ji}(t)$. So if we can estimate $\{I_{jm}^{\text{out}}(t)\}_{1 \le j \ne m \le n, t=1,\cdots,T}$ then with the travel data, we can estimate $\{S_{jm}^{\text{out}}(t)\}_{1 \le j \ne m \le n, t=1,\cdots,T}$. As a result we can then estimate $S_m^{in}(t)$, and $I_m^{in}(t)$.

We discuss the procedure for estimating $I_{jm}^{\text{out}}(t)$. To estimate $I_{jm}^{\text{out}}(t)$ we need to estimate the pandemic situation in country j at day t, i.e. we need to estimate:

$\mathbf{X}_j^+(t) = [S_j^+(t), I_j^+(t), A_j(t), R_j(t), D_j(t), R_j^u(t)]$. Then based on these compartments and the travel data $T_{jm}(t)$, we can estimate $I_{jm}^{\text{out}}(t)$ as $T_{jm}(t) = \frac{T_{jm}(t) I_j^+(t)}{S_j^+(t) + I_j^+(t)}$.

From the global model we have:

$$
\begin{aligned}
U_j(t+1) &= A_j(t+1) + R_j(t+1) + D_j(t+1) \\
&= A_j(t) + Y_{2,j}(t) - Y_{3,j}(t) - Y_{4,j}(t) + R_j(t) + Y_{3,j}(t) + D_j(t) + Y_{4,j}(t) \\
&= A_j(t) + R_j(t) + D_j(t) + Y_{2,j}(t) = U_j(t) + Y_{2,j}(t),
\end{aligned}
$$

so $Y_{2,j}(t) = U_j(t+1) - U_j(t) = \Delta U_j(t)$.

medRxiv preprint doi: https://doi.org/10.1101/2021.04.14.21255465; this version posted April 20, 2021. The copyright holder for this preprint (which was not certified by peer review) is the author/funder, who has granted medRxiv a license to display the preprint in perpetuity.
It is made available under a CC-BY 4.0 International license .

Since $Y_{2,j}(t) \sim \text{Poisson}\big(\gamma_j I_j^+(t-1)\big)$, therefore, we have

$$E(I_j^+(t-1)) = E\big(\frac{\Delta U_j(t)}{\gamma_j}\big). \tag{8}$$

Similarly we also have

$$E(I_j^+(t-2)) = E\big(\frac{\Delta U_j(t-1)}{\gamma_j}\big) \tag{9}$$

From (8) and (9), we have

$$\frac{E(I_j^+(t-1))}{E(I_j^+(t-2))} = \frac{E\big(\frac{\Delta U_j(t)}{\gamma_j}\big)}{E\big(\frac{\Delta U_j(t-1)}{\gamma_j}\big)} = \frac{E(\Delta U_j(t))}{E(\Delta U_j(t-1))} \tag{10}$$

Applying (10) for $t = 1, \cdots, T-1$, we have the sequence of relationships: $\frac{E(I_j^+(1))}{E(I_j(0))} = \frac{E(\Delta U_j(2))}{E(\Delta U_j(1))}$, $\frac{E(I_j^+(2))}{E(I_j^+(1))} = \frac{E(\Delta U_j(3))}{E(\Delta U_j(2))}, \cdots, \frac{E(I_j^+(T-1))}{E(I_j(T-2))} = \frac{E(\Delta U_j(T))}{E(\Delta U_j(T-1))}$. Therefore, based on the available data at country j as $\{A_j(t), R_j(t), D_j(t)\}_{t=1,\cdots,T}$, we can approximate the average realization of the sequence of undetected infected people in country j up to time $(T-1)$ by $I_j(0)$, $I_j^+(1) = I_j(0)\frac{\Delta U_j(2)}{\Delta U_j(1)}$, $I_j^+(2) = I_j^+(1)\frac{\Delta U_j(3)}{\Delta U_j(2)}, \cdots, I_j^+(T-1) = I_j^+(T-2)\frac{\Delta U_j(T)}{\Delta U_j(T-1)}$.

In addition, we also have $R_j^u(t) = R_j^u(t-1) + Y_{5,j}(t-1)$, where $Y_{5,j}(t-1) \sim \text{Poisson}\big(\beta I_j^+(t-1)\big)$. Therefore,

$$E\big(\Delta R_j^u(t-1)\big) = E\big(\beta I_j^+(t-1)\big) \tag{11}$$

We have $R_j(t) = R_j(t-1) + Y_{3,j}(t-1)$, where $Y_{3,j}(t-1) \sim \text{Poisson}\big(\beta A_j(t-1)\big), \forall t = 1, \cdots, T$. Therefore the median value of the sequence $\{\frac{R_j(t)-R_j(t-1)}{A_j(t-1)}\}_{t=1,\cdots,T}$ can be used to approximate β. We denote this median value as $\hat{\beta}$.

The fact $R_j^u(t) = R_j^u(0) + \sum_{i=1}^{t-1} \Delta R_j^u(i)$ and (11) tells us that the average value of $R^u(t)$ at

medRxiv preprint doi: https://doi.org/10.1101/2021.04.14.21255465; this version posted April 20, 2021. The copyright holder for this preprint (which was not certified by peer review) is the author/funder, who has granted medRxiv a license to display the preprint in perpetuity.
It is made available under a CC-BY 4.0 International license .

country j can be reconstructed as

$$\begin{aligned} E\big(R_j^u(t)\big) &= E\big(R_j^u(0)\big) + \sum_{i=1}^{t-1} E(\Delta R_j^u(i)) \\ &= E\big(R_j^u(0)\big) + \sum_{i=1}^{t-1} \beta E\big(I_j^+(i)\big), \end{aligned} \tag{12}$$

where the sequence $\{E\big(I_j^+(i)\big)\}_{j=1,\cdots,T-1}$ and β are estimated as above.

So the average realization of the pandemic at country j which adopts $\{(A_j(t), R_j(t), D_j(t)\}_{t=1,\cdots,T}$ as its active confirmed, recover confirmed and death confirmed can be reconstructed up to time $T-1$ as $\{S_j^+(t), I_j^+(t), A(t), R(t), D(t), R^u(t)\}_{t=1,\cdots,T-1} = \{P_j(t) - U_j(t) - I_j^+(t) - R_j^u(t), I_j^+(t), A(t), R(t), D(t), R_j^u(t)\}_{t=1,\cdots,T-1}$.

This procedure of estimating the average realization of a given country j based on the available data $\{(A_j(t), R_j(t), D_j(t))\}_{t=1,\cdots,T}$ is completed. As a result, this gives us the estimation of $S_{jm}^{\text{out}}(t)$ and $I_{jm}^{\text{out}}(t)$. This means we can estimate $S_m^{in}(t)$, and $I_m^{in}(t)$. Therefore, the underlying true parameter θ_m in a given country m can be approximated marginally by using the above estimating procedure.

We now discuss the proposed distance when estimating θ_m in a given country m by ABC marginally. Following the same argument as in Section 1.4, instead of using the commonly used Euclidean distance to estimate θ_m we first need to standardize each sequence, then we try to learn each parameter under our model assumptions. From the available data $\{(A_m(t), R_m(t), D_m(t))\}_{t=1,\cdots,T}$ of country m, follow the same argument as in Section 1.4, we can add the term

$|\text{median}\{\frac{R_m(t)-R_m(t-1)}{A_m(t-1)}\}_{t=1,\cdots,T} - \text{median}\{\frac{R_m^{(i)}(t)-R_m^{(i)}(t-1)}{A_m^{(i)}(t-1)}\}_{t=1,\cdots,T}|$ to improve the estimation for the recover rate β_m, and adding the term

$|\text{median}\{\frac{D_m(t)-D_m(t-1)}{A_m(t-1)}\}_{t=1,\cdots,T} - \text{median}\{\frac{D_m^{(i)}(t)-D_m^{(i)}(t-1)}{A_m^{(i)}(t-1)}\}_{t=1,\cdots,T}|$ to improve the estimation for the death rate δ_m. We discuss the transmission rate α_m, at a time step $t+1$, we have:

medRxiv preprint doi: https://doi.org/10.1101/2021.04.14.21255465; this version posted April 20, 2021. The copyright holder for this preprint
(which was not certified by peer review) is the author/funder, who has granted medRxiv a license to display the preprint in perpetuity.
It is made available under a CC-BY 4.0 International license .

$S_m(t+1) = S_m^+(t) - Y_{1,m}(t)$, where $Y_{1,m}(\mathbf{X}_m(t)) \sim \text{Poisson}\big(\alpha_m \frac{S_m^+(t)I_m^+(t)}{P_m(t)}\big)$. Therefore, $\alpha_m = P_m(t)E(\frac{S_m^+(t)-S_m(t+1))}{S_m^+(t)I_m^+(t)}\big)$. Notice that $S_m(t+1) = S_m^+(t+1) - S_m^{\text{in}}(t+1) + S_m^{\text{out}}(t+1)$. The hidden states $S_m^+(t)$ and $I_m^+(t)$ can be reconstructed as above or by using the testing argument as above. We discuss the approach by using the testing argument.

From (8) we have: $E(I_m^+(t-1)) = E\big(\frac{\Delta U_m(t)}{\gamma_m}\big)$. Therefore the average realization of $\{I_m^+(t)\}_{t=1,\cdots,T}$ can be reconstructed as $\{\frac{\Delta U_m(t)}{\gamma_m}\}_{t=1,\cdots,T}$. Using (12), we have $E\big(R_m^u(t)\big) = E\big(R_m^u(0)\big) + \sum_{i=1}^{t-1} \beta_m E\big(I_m^+(i)\big)$. Therefore the average realization of $\{R_m^u(t)\}_{t=1,\cdots,T}$ can be reconstructed as $\{R_m^u(0) + \sum_{i=1}^{t-1} \frac{\beta_m \Delta U_m(t)}{\gamma_m}\}_{t=1,\cdots,T}$

The average realization of the pandemic at country m which adopts $\{(A_m(t), R_m(t), D_m(t)\}_{t=1,\cdots,T}$ as its active confirmed, recover confirmed and death confirmed can be reconstructed up to time $T-1$ as $\{S_m^+(t), I_m^+(t), A_m(t), R_m(t), D_m(t), R_m^u(t)\}_{t=1,\cdots,T-1} = \{P_m(t) - U_m(t) - \frac{\Delta U_m(t)}{\gamma_m} - (R_m^u(0) + \sum_{i=1}^{t-1} \frac{\beta_m \Delta U_m(t)}{\gamma_m}), \frac{\Delta U_m(t)}{\gamma_m}, A_m(t), R_m(t), D_m(t), R_m^u(0) + \sum_{i=1}^{t-1} \frac{\beta_m \Delta U_m(t)}{\gamma_m}\}_{t=1,\cdots,T-1}$.

Similarly as above, in step 1 of the ABC algorithm, the parameter $\theta_m^{(i)} = \big(\alpha_m^{(i)}, \beta_m^{(i)}, \delta_m^{(i)}, \gamma_m^{(i)}\big)$ is generated and available to us. If $\gamma_m^{(i)}, \beta_m^{(i)}$ are correctly specified as γ_m, β_m of the underlying true parameter $\theta_m = \big(\alpha_m, \beta_m, \delta_m, \gamma_m\big)$, we would expect the distance

$|\text{median}\{P_m(t)\frac{S_m^+(t)-S_m(t+1))}{S_m^+(t)I_m^+(t)}\}_{1,\cdots,T-1} - \text{median}\{P_m^{(i)}(t)\frac{S_m^{(i)+}(t)-S_m^{(i)}(t+1))}{S_m^{(i)+}(t)I_m^{(i)+}(t)}\}_{1,\cdots,T-1}$ to be close to 0. Where values of the sequence $\{P_m(t)\frac{S_m^+(t)-S_m(t+1))}{S_m^+(t)I_m^+(t)}\}_{1,\cdots,T-1}$ are constructed based on $\gamma_m^{(i)}, \beta_m^{(i)}$ and the available data $\{(A_m(t), R_m(t), D_m(t)\}_{t=1,\cdots,T}$, and values of the sequence $\{P_m^{(i)}(t)\frac{S_m^{(i)+}(t)-S_m^{(i)}(t+1))}{S_m^{(i)+}(t)I_m^{(i)+}(t)}\}_{1,\cdots,T-1}$ are constructed based on $\gamma_m^{(i)}, \beta_m^{(i)}$ and the simulated data $\{(A_m^{(i)}(t), R_m^{(i)}(t), D_m^{(i)}(t)\}_{t=1,\cdots,T}$. So adding the term:

$|\text{median}\{P_m(t)\frac{S_m^+(t)-S_m(t+1))}{S_m^+(t)I_m^+(t)}\}_{1,\cdots,T-1} - \text{median}\{P_m^{(i)}(t)\frac{S_m^{(i)+}(t)-S_m^{(i)}(t+1))}{S_m^{(i)+}(t)I_m^{(i)+}(t)}\}_{1,\cdots,T-1}$ would help to improve the estimation for α_m. Finally, the proposed global distance in the calibrating step of the ABC algorithm is designed as follow:

$\sqrt{\frac{1}{T}\sum_{t=1}^{T}[\big(\frac{A_m^{(i)}(t)-A_m(t)}{\text{sd}(A_m(t))}\big)^2 + \big(\frac{R_m^{(i)}(t)-R_m(t)}{\text{sd}(R_m(t))}\big)^2 + \big(\frac{D_m^{(i)}(t)-D_m(t)}{\text{sd}(D_m(t))}\big)^2]} + \sqrt{d_m},$

medRxiv preprint doi: https://doi.org/10.1101/2021.04.14.21255465; this version posted April 20, 2021. The copyright holder for this preprint (which was not certified by peer review) is the author/funder, who has granted medRxiv a license to display the preprint in perpetuity.
It is made available under a CC-BY 4.0 International license .

where

$$
\begin{aligned}
d_m =& |\text{median}\{\frac{R_m(t)-R_m(t-1)}{A_m(t-1)}\}_{t=1,\cdots,T} - \text{median}\{\frac{R_m^{(i)}(t)-R_m^{(i)}(t-1)}{A_m^{(i)}(t-1)}\}_{t=1,\cdots,T}| \\
&+ |\text{median}\{\frac{D_m(t)-D_m(t-1)}{A_m(t-1)}\}_{t=1,\cdots,T} - \text{median}\{\frac{D_m^{(i)}(t)-D_m^{(i)}(t-1)}{A_m^{(i)}(t-1)}\}_{t=1,\cdots,T}| \\
&+ |\text{median}\{P_m(t)\frac{S_m^{+}(t)-S_m(t+1))}{S_m^{+}(t)I_m^{+}(t)}\}_{1,\cdots,T-1} - \text{median}\{P_m^{(i)}(t)\frac{S_m^{(i)+}(t)-S_m^{(i)}(t+1))}{S_m^{(i)+}(t)I_m^{(i)+}(t)}\}_{1,\cdots,T-1}|.
\end{aligned}
$$

2 Simulation studies

2.1 Simulation 1: Performance of different RABC distance metrics

For our first simulation study, we limit our model to the analysis of only one country, i.e., we only use the internal model. We here demonstrate the impact of the choice of the distance in ABC algorithms and which one to choose in our epidemiological framework.

We simulate $N = 200$ sets of parameters and data, in an ABC fashion, by first simulating a parameter value from the prior and using it to generate data according to the model. We treat these N simulations as our test data set to assess how accurately the true parameters are recovered by ABC using various distance functions. The simulation proceeds as follows.

Step 1. Generating data and parameters: For $i \in \{1, \cdots, N\}$ (N large), we generate the parameter $\theta^{(i)} = (\alpha^{(i)}, \beta^{(i)}, \delta^{(i)}, \gamma^{(i)})$ from uniform priors $\alpha^{(i)} \sim U(0, 2)$, $\beta^{(i)} \sim U(0, 1)$, $\delta^{(i)} \sim U(0, 1)$, and $\gamma^{(i)} \sim U(0, 1)$. Based on the parameters and the stochastic model, we generate a data set Data$^{(i)}$ corresponding to $\theta^{(i)}$. If the generated data set Data$^{(i)}$ satisfies certain conditions making it sufficiently real-world like, such as having the number of confirmed accumulated deaths greater than 1% and lower than 30% of total confirmed cases and having the number of accumulated recovered cases at least twice the number of accumulated deaths, then we keep $\theta^{(i)}$ as a true parameter value to be estimates and treat the generated data $\{A_t^{(i)}, R_t^{(i)}, D_t^{(i)}\}$ as

medRxiv preprint doi: https://doi.org/10.1101/2021.04.14.21255465; this version posted April 20, 2021. The copyright holder for this preprint (which was not certified by peer review) is the author/funder, who has granted medRxiv a license to display the preprint in perpetuity.
It is made available under a CC-BY 4.0 International license .

real observed data. We repeat the process until we obtain 200 underlying true parameter values $\theta^{(i)}$ and the corresponding 200 datasets $\{A_t^{(i)}, R_t^{(i)}, D_t^{(i)}\}$. For simplicity, we fix the initial condition of the six compartments in the model as $\mathbf{X}_1(0) = [S_1(0), I_1(0), A_1(0), R_1(0), D_1(0), R_1^u(0)] = (9999972, 15, 13, 0, 0, 0)$ and set the simulation time period $T = 84$ days for all i.

Step 2. Estimating parameters: For each iteration i, $i \in \{1, \cdots, 200\}$, based on the sequence of $\{A_t^{(i)}, R_t^{(i)}, D_t^{(i)}\}$, we use RABC with different distance metrics to estimate the underlying true parameter value $\theta^{(i)}$. In this estimation step, we choose the acceptance rate 0.01 and sample 1000 particles to form the posterior. From the posterior distribution for each i, we calculate the median values of each parameter: $\hat{\alpha}^{(i)}$, $\hat{\beta}^{(i)}$, $\hat{\delta}^{(i)}$, $\hat{\gamma}^{(i)}$. Then $\hat{\theta}^{(i)} = (\hat{\alpha}^{(i)}, \hat{\beta}^{(i)}, \hat{\delta}^{(i)}, \hat{\gamma}^{(i)})$ is used as the best candidate for estimating the underlying true $\theta^{(i)}$.

Step 3. Evaluating parameter estimates: For each iteration i, $i \in \{1, \cdots, 200\}$, we evaluate estimation accuracy in terms of the absolute bias, absolute relative bias, interquartile range, and coverage rate of the interquartile for each parameter $\alpha^{(i)}, \beta^{(i)}, \delta^{(i)}, \gamma^{(i)}$ and its average. These accuracy measurements are defined as follows. For a given parameter $\alpha^{(i)}$ the absolute bias is defined as $|\hat{\alpha}^{(i)} - \alpha^{(i)}|$, and the absolute relative bias is defined as $|\frac{\hat{\alpha}^{(i)} - \alpha^{(i)}}{\alpha^{(i)}}|$. Similarly for $\beta^{(i)}$, $\gamma^{(i)}$, and $\delta^{(i)}$. Average absolute bias for all parameters is defined as $(|\hat{\alpha}^{(i)} - \alpha^{(i)}| + |\hat{\beta}^{(i)} - \beta^{(i)}| + |\hat{\delta}^{(i)} - \delta^{(i)}| + |\hat{\gamma}^{(i)} - \gamma^{(i)}|)/4$ and average absolute relative bias for all parameters is defined as $(|\frac{\hat{\alpha}^{(i)} - \alpha^{(i)}}{\alpha^{(i)}}| + |\frac{\hat{\beta}^{(i)} - \beta^{(i)}}{\beta^{(i)}}| + |\frac{\hat{\delta}^{(i)} - \delta^{(i)}}{\delta^{(i)}}| + |\frac{\hat{\gamma}^{(i)} - \gamma^{(i)}}{\gamma^{(i)}}|)/4$. For each parameter, we also calculate the interquartile range (IQR) of the posterior, denoted IQR$^{(i)}$, which is the difference between the third and the first quartile of the resulting ABC posterior distribution. Furthermore, we check whether the IQR of the posterior covers the underlying true parameter value, which we use to calculate the coverage rate CR$^{(i)}$. Then the average of the IQR for all four parameters and the average of the coverage rate is calculated to characterize the overall performance of the two distance metrics. Finally, the average over the 200 iterations of these accuracy metrics is calculated, which we use as our overall accuracy metrics for comparing the performance of the

medRxiv preprint doi: https://doi.org/10.1101/2021.04.14.21255465; this version posted April 20, 2021. The copyright holder for this preprint (which was not certified by peer review) is the author/funder, who has granted medRxiv a license to display the preprint in perpetuity.
It is made available under a CC-BY 4.0 International license .

two RABC distance metrics.

Table S1 summarizes the different prediction accuracy measures for the two distances. This table shows that the distance we proposed increases the estimation accuracy in terms of relative bias. The two types of bias are much smaller compared to using Euclidean distance. We also observe that the IQR for the proposed distance is higher than the IQR for the Euclidean distance, but the proposed distance also yields more narrow IQR. This means that our proposed distance metric more frequently correctly bounds the true parameter values. The IQR is about 2.5 times smaller when using the proposed distance metric instead of Euclidean distance. Figure S1 shows boxplots of the IQR for the two distance metrics.

Table S1: Accuracy of Euclidean distance and our proposed distance when using RABC to estimate the four parameters of the local model.

Accuracy	Distances	Average	alpha	beta	delta	gamma
Absolute bias	Euclidean	0.071	0.125	0.046	0.012	0.101
	Proposed	0.028	0.054	0.004	0.002	0.052
Absolute relative bias	Euclidean	0.148	0.107	0.089	0.093	0.303
	Proposed	0.077	0.039	0.007	0.022	0.241
IQR	Euclidean	0.153	0.264	0.094	0.031	0.222
	Proposed	0.060	0.107	0.014	0.012	0.109
IQ coverage	Euclidean	0.677	0.620	0.675	0.770	0.645
	Proposed	0.777	0.635	0.880	0.960	0.635

medRxiv preprint doi: https://doi.org/10.1101/2021.04.14.21255465; this version posted April 20, 2021. The copyright holder for this preprint
(which was not certified by peer review) is the author/funder, who has granted medRxiv a license to display the preprint in perpetuity.
It is made available under a CC-BY 4.0 International license .

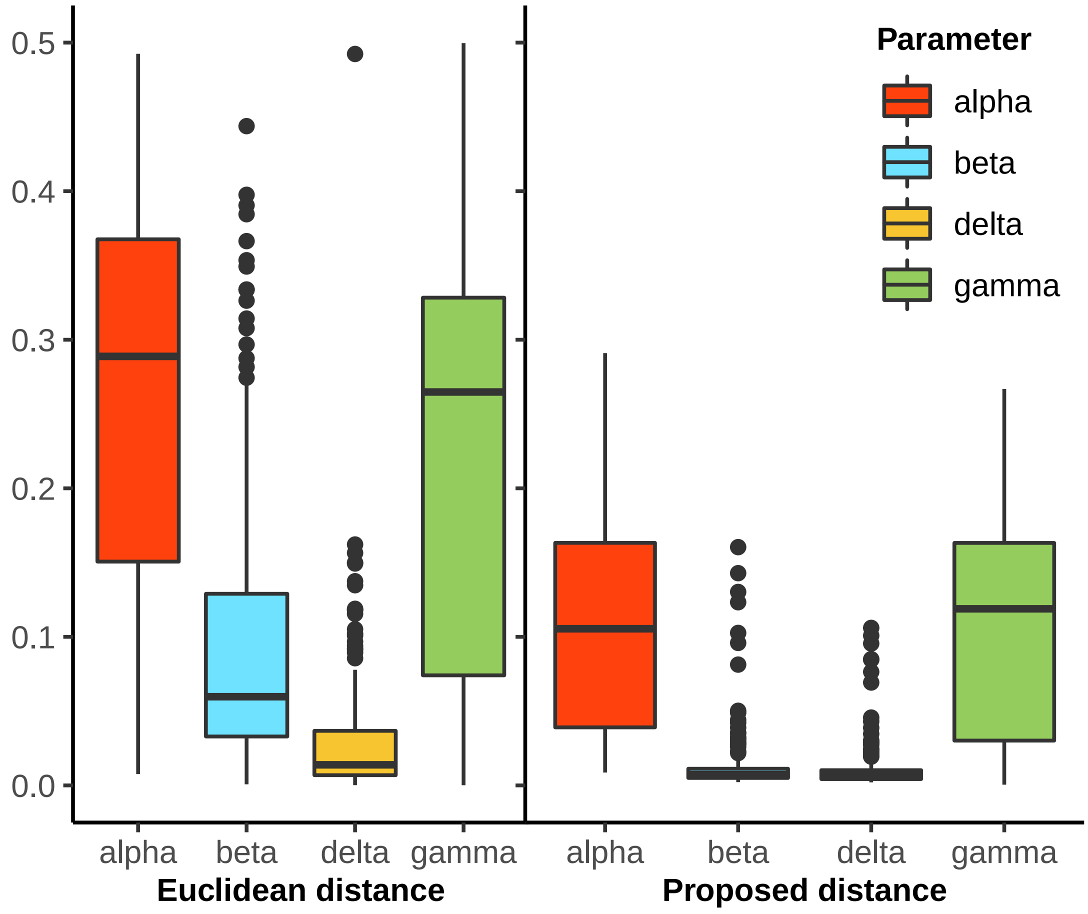

Figure S1: Interquartile range of the posterior for parameters estimation of the proposed distance and the Euclidean distance for the local model of one country.

medRxiv preprint doi: https://doi.org/10.1101/2021.04.14.21255465; this version posted April 20, 2021. The copyright holder for this preprint
(which was not certified by peer review) is the author/funder, who has granted medRxiv a license to display the preprint in perpetuity.
It is made available under a CC-BY 4.0 International license .

2.2 Simulation 2: Performance of local and global estimation

In this simulation study, we investigate the accuracy of three different estimation procedures for the global travel model consisting of three countries. (We limit this investigation to three countries for simplicity.) The first procedure uses a local approach with the Euclidean distance to estimate each country's parameters independently and ignores the travel between the countries. We call this estimation procedure Euclidean local, and we use it as a benchmark to be compared with the other two approaches. Then we consider a global estimation procedure as discussed in Section 1.5 to estimate each country's parameters. Here we use two distance metrics, Euclidean distance and the distance proposed in Section 1.5. We call these estimation procedures Euclidean global and Proposed global, respectively. The simulation is set up as follows.

Step 1. Generating data and parameters: For $i \in \{1, \cdots, N\}$ (N large), we generate the parameter $\theta^{(i)} = (\theta_1^{(i)}, \theta_2^{(i)}, \theta_3^{(i)})$, where for each j in 1, 2, 3, $\theta_j^{(i)} = (\alpha_j^{(i)}, \beta_j^{(i)}, \delta_j^{(i)}, \gamma_j^{(i)})$ from uniform priors as $\alpha_j^{(i)} \sim U(0,2)$, $\beta_j^{(i)} \sim U(0,1)$, $\delta_j^{(i)} \sim U(0,1)$, and $\gamma_j^{(i)} \sim U(0,1)$. Based on the parameters and the stochastic model, we generate a data set Data$^{(i)}$ corresponding to $\theta^{(i)}$. If the generated data set Data$^{(i)}$ satisfies the conditions described above for Simulation 1, we retain $\theta^{(i)}$ and treat it as the underlying true parameter value; we also retain the data $\{A_{(t,j)}^{(i)}, R_{(t,j)}^{(i)}, D_{(t,j)}^{(i)}\}$, for $j = 1, 2, 3$, and treat them as the observed data from these three countries. We repeat the procedure until we have 500 parameter values and their corresponding data sets $\{A_{(t,j)}^{(i)}, R_{(t,j)}^{(i)}, D_{(t,j)}^{(i)}\}_{j=1,2,3}$.

For simplicity, we fix the initial condition of the six compartments in the model as

$$\mathbf{X}_1(0) = [S_1(0), I_1(0), A_1(0), R_1(0), D_1(0), R_1^u(0)] = (9999720, 150, 130, 0, 0, 0),$$

$$\mathbf{X}_2(0) = [S_2(0), I_2(0), A_2(0), R_2(0), D_2(0), R_2^u(0)] = (2999970, 20, 10, 0, 0, 0),$$

$$\mathbf{X}_3(0) = [S_3(0), I_3(0), A_3(0), R_3(0), D_3(0), R_3^u(0)] = (1999970, 15, 15, 0, 0, 0),$$

medRxiv preprint doi: https://doi.org/10.1101/2021.04.14.21255465; this version posted April 20, 2021. The copyright holder for this preprint (which was not certified by peer review) is the author/funder, who has granted medRxiv a license to display the preprint in perpetuity.
It is made available under a CC-BY 4.0 International license .

and set the simulation period $T = 84$ days for all i. Each day, the number of outbound travelers from country j, $j = 1, 2, 3$, is drawn from a normal distribution with mean $\mu_j = P_j * 0.0003$ and standard deviation $\text{sd}_j = 0.05 * \mu_j$, where P_j is the size of the population of country j. Those outbound travelers will enter one of the neighboring countries with proportions that are proportional to the sizes (populations) of the target countries. For example, if there are n_1 people leaving country 1, the number of them entering country 2 is $\frac{n_1 P_2}{P_2+P_3}$ and the number of them entering country 3 is $\frac{n_1 P_3}{P_2+P_3}$.

Step 2. Estimating parameters: For each iteration i, $i \in \{1, \cdots, 500\}$, based on the sequence of $\{A^{(i)}_{(t,j)}, R^{(i)}_{(t,j)}, D^{(i)}_{(t,j)}\}_{j=1,2,3}$, we first naively use RABC with the local estimation approach and Euclidean distance to estimate $\theta^{(i)}$. Then we use RABC with the global estimation approach with the two distance metrics to estimate the underlying true parameters $\theta^{(i)}$. Then $\hat{\theta}^{(i)}_j = (\hat{\alpha}^{(i)}_j, \hat{\beta}^{(i)}_j, \hat{\delta}^{(i)}_j, \hat{\gamma}^{(i)}_j)$ is obtained as the median of the RABC posterior samples and is used to estimate the underlying true $\theta^{(i)}_j$, for $j = 1, 2, 3$.

Step 3. Evaluating parameter estimates: For each iteration i, $i \in \{1, \cdots, 500\}$, for each country, we evaluate the accuracy of our parameter estimates based on the absolute bias, absolute relative bias, interquartile range (IQR), and coverage rate of IQR for each parameter $\alpha^{(i)}, \beta^{(i)}, \delta^{(i)}, \gamma^{(i)}$ and its average as in simulation 1. The final accuracy measurements are calculated by averaging the accuracy measurements across all three countries. When averaging accuracy measures over multiple countries, we consider two weighted averages, one having equal weights for all countries regardless of their population sizes and the other weighted based on relative population sizes. In the latter, the weights are $\frac{P_1}{P_1+P_2+P_3}$ for country 1, $\frac{P_2}{P_1+P_2+P_3}$ for country 2, and $\frac{P_3}{P_1+P_2+P_3}$ for country 3.

Tables S2 and S3 show the overall accuracy of different estimation procedures using equal weights for each country (Table S2) and using population-based weights for each country (Table S3). The two tables convey the same message: using a local approach to estimate the parameters

medRxiv preprint doi: https://doi.org/10.1101/2021.04.14.21255465; this version posted April 20, 2021. The copyright holder for this preprint (which was not certified by peer review) is the author/funder, who has granted medRxiv a license to display the preprint in perpetuity.
It is made available under a CC-BY 4.0 International license .

Table S2: Accuracy of Euclidean distance and our proposed distance when using RABC to estimate the four parameters of the global model. For simplicity, we consider a small world of just three countries with different population sizes; here each country has the same weight when computing the overall accuracy.

Accuracy	Estimation procedure	Average	alpha	beta	delta	gamma
Absolute bias	Euclidean local	0.496	1.328	0.108	0.058	0.490
	Euclidean global	0.236	0.448	0.070	0.030	0.397
	Proposed global	0.205	0.497	0.023	0.054	0.248
Absolute relative bias	Euclidean local	0.928	1.611	0.176	0.591	1.332
	Euclidean global	0.502	0.534	0.109	0.280	1.085
	Proposed global	0.494	0.629	0.034	0.545	0.769
IQR range	Euclidean local	0.171	0.263	0.179	0.060	0.180
	Euclidean global	0.149	0.218	0.129	0.040	0.210
	Proposed global	0.091	0.135	0.041	0.038	0.151
IQ coverage	Euclidean local	0.528	0.463	0.577	0.690	0.381
	Euclidean global	0.610	0.539	0.649	0.714	0.540
	Proposed global	0.631	0.543	0.647	0.761	0.574
95% range	Euclidean local	0.410	0.646	0.406	0.157	0.430
	Euclidean global	0.361	0.542	0.309	0.107	0.486
	Proposed global	0.247	0.378	0.116	0.115	0.380
95% coverage	Euclidean local	0.866	0.819	0.956	0.978	0.712
	Euclidean global	0.935	0.893	0.957	0.980	0.911
	Proposed global	0.958	0.923	0.972	0.989	0.949

medRxiv preprint doi: https://doi.org/10.1101/2021.04.14.21255465; this version posted April 20, 2021. The copyright holder for this preprint (which was not certified by peer review) is the author/funder, who has granted medRxiv a license to display the preprint in perpetuity.
It is made available under a CC-BY 4.0 International license .

Table S3: Accuracy of Euclidean distance and our proposed distance when using RABC to estimate the four parameters of the global model. For simplicity, we consider a small world of just three countries with different population sizes; here each country's contribution to the overall accuracy is weighted based on the size of its population.

Accuracy	Estimation procedure	Average	alpha	beta	delta	gamma
Absolute bias	Euclidean local	0.380	0.939	0.095	0.048	0.438
	Euclidean global	0.194	0.338	0.064	0.025	0.351
	Proposed global	0.155	0.372	0.019	0.043	0.186
Absolute relative bias	Euclidean local	0.862	1.277	0.162	0.636	1.371
	Euclidean global	0.512	0.459	0.101	0.304	1.185
	Proposed global	0.414	0.583	0.028	0.564	0.480
IQR range	Euclidean local	0.147	0.228	0.156	0.048	0.154
	Euclidean global	0.131	0.191	0.118	0.035	0.182
	Proposed global	0.073	0.111	0.033	0.030	0.120
IQR coverage	Euclidean local	0.526	0.468	0.575	0.661	0.400
	Euclidean global	0.610	0.548	0.641	0.701	0.550
	Proposed global	0.613	0.536	0.595	0.733	0.586
95% range	Euclidean local	0.360	0.571	0.361	0.128	0.380
	Euclidean global	0.325	0.488	0.284	0.094	0.433
	Proposed global	0.201	0.310	0.094	0.090	0.309
95% coverage	Euclidean local	0.860	0.812	0.950	0.973	0.704
	Euclidean global	0.928	0.887	0.954	0.976	0.898
	Proposed global	0.948	0.905	0.962	0.985	0.941

medRxiv preprint doi: https://doi.org/10.1101/2021.04.14.21255465; this version posted April 20, 2021. The copyright holder for this preprint
(which was not certified by peer review) is the author/funder, who has granted medRxiv a license to display the preprint in perpetuity.
It is made available under a CC-BY 4.0 International license .

in the travel model is not appropriate. As shown in these tables, the Euclidean local estimation procedure yields the highest bias, largest interquartile range, largest 95 $\%$ percentile range, and lowest coverage. The performance is better for the Euclidean global procedure. As expected, the proposed distance, which takes into account the travel model, performs best of the three.

2.3 Simulation 3: Effectiveness of travel regulation

In this simulation study, we study the effectiveness of different travel regulation policies. We compare the percentages of people allowed to travel under each policy and the pandemic situation in the country adopting the policy. The simulation is set up as follows.

Step 1. Generating data and parameters: For $i \in \{1, \cdots, N\}$ (N large), we generate the parameter $\theta^{(i)} = (\theta_1^{(i)}, \theta_2^{(i)}, \theta_3^{(i)}, \theta_4^{(i)})$, where for each j in 1, 2, 3, 4 $\theta_j^{(i)} = (\alpha_j^{(i)}, \beta_j^{(i)}, \delta_j^{(i)}, \gamma_j^{(i)})$, from uniform priors as $\alpha_j^{(i)} \sim U(\epsilon, 1-\epsilon)$, $\beta_j^{(i)} \sim U(\epsilon, 0.25-\epsilon)$, $\delta_j^{(i)} \sim U(\epsilon, 0.25-\epsilon)$, and $\gamma_j^{(i)} \sim U(\epsilon, 1-\epsilon)$. We chose $\epsilon = 0.001$ to make sure that the generated parameters do not fall at the boundaries of the priors and cause the generation of atypical data. We also added some constraints to ensure the parameter values are reasonable by only keeping parameters with $R_0 = \frac{\alpha_j^{(i)}}{\beta_j^{(i)}+\gamma_j^{(i)}}$ between 0.47 and 6.47 as reported for different regions around the world (*11*). To investigate the effectiveness of travel regulations, we use one more constraint to set the reproduction number R_0 in these 4 countries in 4 different zones, where country 1 has R_0 between 0.47 and 0.9 , country 2 has R_0 between 0.9 and 1, country 3 has R_0 between 1 and 1.1, and country 4 has R_0 between 1.1 and 6.47. The initial conditions of each country are generated randomly as $(S_j^{(i)}(0), I_j^{(i)}(0), A_j^{(i)}(0), R_j^{(i)}(0), D_j^{(i)}(0), R_j^{u(i)}(0)) = (P_j - (I_j^{(i)}(0) + I_j^{(i)}(0)), I_j^{(i)}(0), A_j^{(i)}(0), 0, 0, 0)$, where $P_i \sim U(50 * 10^4, 100 * 10^6)), I_j^{(i)}(0) \sim U(0, 200), A_j^{(i)}(0) \sim U(0, 10)$. Based on the parameters and the stochastic model, we generate a data set Data$^{(i)}$ corresponding to $\theta^{(i)}$. If the generated data set Data$^{(i)}$ satisfies the conditions described for Simulation 1 above, we keep $\theta^{(i)}$ and treat it as the underlying true value of the

medRxiv preprint doi: https://doi.org/10.1101/2021.04.14.21255465; this version posted April 20, 2021. The copyright holder for this preprint (which was not certified by peer review) is the author/funder, who has granted medRxiv a license to display the preprint in perpetuity.
It is made available under a CC-BY 4.0 International license .

parameter; we also retain the data $\{A^{(i)}_{(t,j)}, R^{(i)}_{(t,j)}, D^{(i)}_{(t,j)}\}$ for $j = 1, 2, 3, 4$, which we treat as the observed data collected from each country. We keep generating data till we get 200 underlying true parameters $\theta^{(i)}$ and the corresponding 200 data sets $\{A^{(i)}_{(t,j)}, R^{(i)}_{(t,j)}, D^{(i)}_{(t,j)}\}_{j=1,2,3,4}$. We fix the duration of the simulation to $T = 42$ days for all i. Each day, the total number of of outbound travelers from country j, $j = 1, 2, 3$, is drawn from a normal distribution with mean $\mu^{(i)}_j = P^{(i)}_j * 0.0003$ and standard deviation $\text{sd}^{(i)}_j = 0.05 * \mu^{(i)}_j$, where $P^{(i)}_j$ is the size of the population of country j. The outbound travelers enter other countries in proportion to sizes of their populations.

Step 2. Estimation step: For each iteration i, $i \in \{1, \cdots, 200\}$, based on the sequence of $\{A^{(i)}_{(t,j)}, R^{(i)}_{(t,j)}, D^{(i)}_{(t,j)}\}_{j=1,2,3,4}$, we use the proposed global approach to estimate the underlying true $\theta^{(i)}$. Then $\hat{\theta}^{(i)}_j = (\hat{\alpha}^{(i)}_j, \hat{\beta}^{(i)}_j, \hat{\delta}^{(i)}_j, \hat{\gamma}^{(i)}_j)$ are obtained as the median values of the RABC posterior samples and are used to estimate the underlying true $\theta^{(i)}_j$, for $j = 1, 2, 3, 4$.

Step 3. Prediction step: For each iteration i, $i \in \{1, \cdots, 200\}$, based on the estimated parameter $\hat{\theta}^{(i)}$, we simulate data for the following two weeks under eight different travel regulation policies. The first two are the most extreme, where all countries are either fully open or fully closed. The third and the fourth are currently used policies, where a 14-days quarantine is required for all arrivals or a 14-day quarantine is required required for arrivals from high-risk countries only. The last 4 policies are our proposed travel regulation policies. We describe each policy in detail below.

P-1 All countries are fully open and allow all airline travel as usual.

P-2 All countries are fully closed and no airline travel is allowed across their borders.

P-3 The country requires a 14-day quarantine for all arrivals. This policy is currently used in many countries such as Korea or India. The other countries are fully open.

medRxiv preprint doi: https://doi.org/10.1101/2021.04.14.21255465; this version posted April 20, 2021. The copyright holder for this preprint (which was not certified by peer review) is the author/funder, who has granted medRxiv a license to display the preprint in perpetuity.
It is made available under a CC-BY 4.0 International license .

P-4 The country requires a 14-day quarantine for travelers from high-risk countries only, i.e., countries with the average number of active confirmed daily cases greater than 20 in 100000 people during the last 2 weeks, and no quarantine for arrivals from other countries. P-4 is a more flexible policy that is currently used by the UK. The other countries are fully open.

P-5 The country adopts a simplified version of the proposed average control policy: we regulate travel such that the average number of daily undetected infected cases is at most 10% higher than the maximum number of daily cases under P-2. The other countries are fully open.

P-6 The country adopts the proposed probability control policy: we regulate travel such that the average number of daily undetected infected cases is at most 10% higher than the maximum number of daily cases under P-2 with probability at least 90%. The other countries are fully open.

P-7 Policy 7 is similar to P-5 but we use the full version of the proposed average control policy as in Example 1 of Section 1.3. The other countries are fully open.

P-8 Policy 8 is similar to P-6 but we use the full version of the proposed probability control policy as in Example 1 of Section 1.3. The other countries are fully open.

Policy effectiveness is evaluated based on two factors: the percentage of people allowed to travel and the pandemic situation in the country once the policy is adopted.

1. The percentage of people allowed to enter the country under each policy is denoted Tc. This number is calculated using the number of people allowed to travel inbound to the country divided by the total number of people willing to enter the country.

medRxiv preprint doi: https://doi.org/10.1101/2021.04.14.21255465; this version posted April 20, 2021. The copyright holder for this preprint (which was not certified by peer review) is the author/funder, who has granted medRxiv a license to display the preprint in perpetuity.
It is made available under a CC-BY 4.0 International license .

2. The percentage of people that will travel due to each policy is denoted Te. This number is an adjusted version Tc. If a 14-day quarantine is applied to a country, we assume that only 5% of the normal number of travelers from this country are willing to travel under this policy. The choice of 5% is based on the data provided by Korea Tourism Organization. (Korea is one of the countries that require a 14-day quarantine for all arrivals.) This adjustment gives us more insights concerning the effect of the 14-day quarantine requirement. After this adjustment, the percentage of expected inbound travelers is obtained by using the number of expected inbound travelers divided by the normal number of inbound travelers.

The effectiveness of policies on the epidemic in the considered country is evaluated based on 7 factors.

1. Percentage of active confirmed imported cases that enter the country due to each policy. This number is calculated using the total number of inbound traveling active confirmed cases that eventually become active confirmed cases, divided by the total number of inbound travelers during the regulation period. We denote this category as IA.

2. Percentage of undetected infected imported cases entering the country due to each policy. This number is obtained using the total number of undetected infected cases traveling inbound divided by the total number of inbound travelers during the regulation period. We denote this category as II.

3. Percentage of undetected infected imported cases when quarantining after entering the country. A policy that does not require quarantine is equivalent to a 0 -day quarantine. This number is obtained by taking the total number of undetected infected inbound travelers after quarantine divided by the total number of inbound travelers during the regulation period. We denote this category as IIQ.

medRxiv preprint doi: https://doi.org/10.1101/2021.04.14.21255465; this version posted April 20, 2021. The copyright holder for this preprint (which was not certified by peer review) is the author/funder, who has granted medRxiv a license to display the preprint in perpetuity.
It is made available under a CC-BY 4.0 International license .

4. Relative change in total new cases (detected and undetected), denoted as RU. This number is calculated as the difference in the total number of cases at the end of the regulation period and the beginning of the regulation period, divided by the total number of cases at the beginning of the regulation period.

5. Relative change in total new active confirmed cases, denoted as RA. This number is calculated similarly to RU above but instead of using the number of cases, all counts are based on the number of active confirmed cases.

6. Percent change in total new cases, denoted as PU. This number is calculated as the difference in the total number of cases at the end of the regulation period and the beginning of the regulation period, divided by the population of the country.

7. Percent change in total confirmed cases, denoted as PA. This number is calculated as the difference in the total number of confirmed cases at the end of the regulation period and the beginning of the regulation period, divided by the population of the country.

We generate 1000 stochastic realizations conditional on the estimated parameters and the estimated initial conditions at the beginning of the regulation period. For each realization, we calculate the above metrics, and we report the 0.025 and 0.975 percentile values of each based on the 1000 realizations. To give a fair judgment on the effectiveness of travel regulation on the pandemic, we stratify the metrics by dividing countries to three different groups, where Group 1 corresponds to countries with an effective reproduction number $R(t)$ lower than 0.9, group 2 corresponds to countries with $R(t)$ between 0.9 and 1.1, and Group 3 for countries with an R_0 greater than 1.1. Notice that for our model, following Diekmann et al. (2009) (*12*), we can show that the effective reproduction number $R_i(t)$ of a given country i is $R_i(t) = \frac{S_i^{(t)+}\alpha_i}{P_i(t)(\beta_i+\gamma_i)}$. The overall average across these 200 iterations of the above metrics for each group of countries is calculated and used as the final measurement to compare the effectiveness of different policies.

medRxiv preprint doi: https://doi.org/10.1101/2021.04.14.21255465; this version posted April 20, 2021. The copyright holder for this preprint (which was not certified by peer review) is the author/funder, who has granted medRxiv a license to display the preprint in perpetuity.
It is made available under a CC-BY 4.0 International license .

In Table S5, we see that under P-4 the number of expected inbound travelers, Te, is higher than P-5, the simplified version of our proposed average control policy. However, under P-4, the percent of undetected infected after done with quarantine, IIQ enter countries of Group 1 is about $(0.03\%, 0.03\%)$ and Group 2 is about $(0.03\%, 0.04\%)$. These values quite high compare to $(0.00\%, 0.00\%)$ for both groups as in P-5. This is because the average of increased cases each day in the last 14 days was used to decide which countries belong to a green zone or red zone. However, the number of undetected infectious cases may grow very fast in the green zone countries, and in the absence of quarantine, undetected infectious cases from green zone countries may spread the disease fast in the arrival country.

2.4 Simulation 4. Effectiveness of policy coordination

In this simulation study, we study the effectiveness of a global response on the pandemic in terms of the percentage of people allowed to travel and the overall worldwide pandemic situation under a coordinated policy. Simulations are set up similarly to those in Section 2.3 but here we consider a world of 8 countries, where countries 1 and 2 with $R(0)$ greater between 1.1 and 6.47, countries 3 and 4 with $R(0)$ between 1 and 1.1, countries 5 and 6 with $R(0)$ between 0.9 and 1, and countries 7 and 8 with $R(0)$ from 0.47 to 0.9.

We consider 8 different policy coordination scenarios:

S-1 All countries are fully open and allow all travel.

S-2 All countries are fully closed and do not allow any airline travel .

S-3 All countries require a 14-day quarantine for all arrivals.

S-4 All countries use the simplified version of the proposed average control policy.

medRxiv preprint doi: https://doi.org/10.1101/2021.04.14.21255465; this version posted April 20, 2021. The copyright holder for this preprint (which was not certified by peer review) is the author/funder, who has granted medRxiv a license to display the preprint in perpetuity.
It is made available under a CC-BY 4.0 International license .

S-5 Countries 1, 3, 5, 7 require a 14-day quarantine for all arrivals, and countries 2, 4, 6, 8 allow no inbound travel.

S-6 Countries 1, 3, 5, 7 use the simplified version of the proposed average control policy, and countries 2, 4, 6, 8 allow no inbound travel.

S-7 Countries 1, 3, 5, 7 require a 14-day quarantine for all arrivals, and countries 2, 4, 6, 8 are fully open.

S-8 Countries 1, 3, 5, 7 use the simplified version of the proposed average control policy, and countries 2, 4, 6, 8 are fully open.

The coordination effectiveness is evaluated based on the overall change in the global pandemic and for each group of countries as in the simulation studies of Section 2.3.

medRxiv preprint doi: https://doi.org/10.1101/2021.04.14.21255465; this version posted April 20, 2021. The copyright holder for this preprint (which was not certified by peer review) is the author/funder, who has granted medRxiv a license to display the preprint in perpetuity.
It is made available under a CC-BY 4.0 International license .

Table S4: We show (0.025, 0.975) percentiles of pandemic changes for different scenarios. For a given policy, the upper value and lower value of each measurement are the 0.025 percentile value and the 0.975 percentile value, respectively. G1, G2, G3 denotes countries in Group 1, 2, and 3, respectively. RU is the relative change in number of cases (including detected and undetected), RA is the relative change in number of cases that were confirmed, PU is the percent change in total new cases, and PA is the percent change in total new confirmed cases.

		P-1	P-2	P-3	P-4	P-5	P-6	P-7	P-8
G1	RU	2.53	0.06	0.64	0.88	0.06	0.06	0.11	0.06
		3.20	0.27	0.92	1.26	0.27	0.26	0.35	0.26
	RA	1.58	0.08	0.86	0.99	0.08	0.08	0.12	0.08
		2.14	0.27	1.15	1.36	0.27	0.27	0.34	0.27
	PU (%)	0.00	0.00	0.00	0.00	0.00	0.00	0.00	0.00
		0.00	0.00	0.00	0.00	0.00	0.00	0.00	0.00
	PA (%)	0.00	0.00	0.00	0.00	0.00	0.00	0.00	0.00
		0.00	0.00	0.00	0.00	0.00	0.00	0.00	0.00
G2	RU	1.50	0.45	0.63	0.86	0.46	0.45	0.48	0.45
		2.05	0.84	1.02	1.32	0.84	0.84	0.88	0.84
	RA	0.99	0.36	0.60	0.71	0.37	0.36	0.39	0.36
		1.37	0.64	0.90	1.04	0.64	0.64	0.67	0.64
	PU (%)	0.02	0.01	0.01	0.01	0.01	0.01	0.01	0.01
		0.02	0.02	0.02	0.02	0.02	0.02	0.02	0.02
	PA (%)	0.01	0.01	0.01	0.01	0.01	0.01	0.01	0.01
		0.02	0.01	0.01	0.01	0.01	0.01	0.01	0.01
G3	RU	6.28	6.30	6.28	6.28	6.28	6.28	6.28	6.28
		6.65	6.67	6.65	6.65	6.65	6.65	6.65	6.65
	RA	5.32	5.33	5.32	5.32	5.32	5.32	5.32	5.32
		5.56	5.57	5.56	5.56	5.56	5.56	5.56	5.56
	PU (%)	5.39	5.40	5.38	5.39	5.38	5.38	5.38	5.38
		5.50	5.52	5.50	5.50	5.50	5.50	5.50	5.50
	PA (%)	2.39	2.40	2.39	2.39	2.39	2.39	2.39	2.39
		2.45	2.46	2.45	2.45	2.45	2.45	2.45	2.45

medRxiv preprint doi: https://doi.org/10.1101/2021.04.14.21255465; this version posted April 20, 2021. The copyright holder for this preprint (which was not certified by peer review) is the author/funder, who has granted medRxiv a license to display the preprint in perpetuity.
It is made available under a CC-BY 4.0 International license .

Table S5: We show (0.025, 0.975) percentiles of travel effects for different policies. For a given policy, the upper value and lower value of each measurement are the 0.025 percentile value and the 0.975 percentile value, respectively. IA is the percentage among the incoming travellers that will eventually become active confirmed after arrival, II is the percentage among the incoming travellers that are undetected infectious, IIQ is the percentage of the incoming travellers who are undetected infectious after the quarantine if the destination country requires a 14-day quarantine, Tc is the percentage of inbound travel capacity, and Te is the percentage of expected of inbound travel.

		P-1	P-2	P-3	P-4	P-5	P-6	P-7	P-8
G1	IA (%)	0.09	0.00	0.09	0.09	0.00	0.00	0.00	0.00
		0.11	0.00	0.11	0.11	0.00	0.00	0.01	0.00
	II (%)	0.17	0.00	0.17	0.17	0.00	0.00	0.00	0.00
		0.19	0.00	0.19	0.19	0.00	0.00	0.01	0.00
	IIQ (%)	0.17	0.00	0.00	0.03	0.00	0.00	0.00	0.00
		0.19	0.00	0.00	0.03	0.00	0.00	0.01	0.00
	Tc	100%	0%	100%	100%	34%	0%	37%	0%
	Te	100%	0%	5%	89%	34%	0%	37%	0%
G2	IA (%)	0.09	0.00	0.09	0.09	0.00	0.00	0.00	0.00
		0.11	0.00	0.11	0.11	0.00	0.00	0.01	0.00
	II (%)	0.18	0.00	0.18	0.18	0.00	0.00	0.01	0.00
		0.20	0.00	0.20	0.20	0.00	0.00	0.02	0.00
	IIQ (%)	0.18	0.00	0.00	0.03	0.00	0.00	0.01	0.00
		0.20	0.00	0.00	0.04	0.00	0.00	0.02	0.00
	Tc	100%	0%	100%	100%	60%	0%	63%	0%
	Te	100%	0%	5%	89%	60%	0%	63%	0%
G3	IA (%)	0.00	0.00	0.00	0.00	0.00	0.00	0.00	0.00
		0.00	0.00	0.00	0.00	0.00	0.00	0.00	0.00
	II (%)	0.00	0.00	0.00	0.00	0.00	0.00	0.00	0.00
		0.00	0.00	0.00	0.00	0.00	0.00	0.00	0.00
	IIQ (%)	0.00	0.00	0.00	0.00	0.00	0.00	0.00	0.00
		0.00	0.00	0.00	0.00	0.00	0.00	0.00	0.00
	Tc	100%	0%	100%	100%	34%	0%	34%	0%
	Te	100%	0%	5%	100%	34%	0%	34%	0%

medRxiv preprint doi: https://doi.org/10.1101/2021.04.14.21255465; this version posted April 20, 2021. The copyright holder for this preprint
(which was not certified by peer review) is the author/funder, who has granted medRxiv a license to display the preprint in perpetuity.
It is made available under a CC-BY 4.0 International license .

Table S6: We show (0.025, 0.975) percentile of pandemic changes for different scenarios. For a given scenario, the upper value and lower value of each measurement are the 0.025 percentile value and the 0.975 percentile value, respectively. G denotes all countries. See Table S4 caption for more information.

		S-1	S-2	S-3	S-4	S-5	S-6	S-7	S-8
G	RU	10.68	2.65	4.02	2.66	3.45	2.65	6.79	6.01
		11.56	3.06	4.51	3.07	3.92	3.07	7.46	6.61
	RA	8.13	2.77	4.92	2.77	4.08	2.77	6.34	5.04
		8.89	3.13	5.43	3.14	4.55	3.13	6.97	5.56
	PU (%)	8.35	8.31	8.28	8.31	8.29	8.31	8.31	8.33
		8.39	8.35	8.32	8.35	8.33	8.35	8.35	8.37
	PA (%)	4.40	4.38	4.37	4.38	4.37	4.38	4.38	4.39
		4.42	4.40	4.39	4.40	4.39	4.40	4.41	4.41
G1	RU	11.16	0.59	3.17	0.60	1.84	0.59	7.24	6.01
		12.23	0.93	3.61	0.94	2.21	0.93	7.99	6.70
	RA	9.01	0.74	4.42	0.75	2.52	0.74	6.62	4.85
		9.95	1.06	4.90	1.07	2.91	1.07	7.33	5.48
	PU (%)	0.05	0.01	0.02	0.01	0.01	0.01	0.03	0.03
		0.05	0.01	0.02	0.01	0.01	0.01	0.04	0.03
	PA (%)	0.03	0.00	0.01	0.00	0.01	0.00	0.02	0.02
		0.03	0.00	0.02	0.00	0.01	0.00	0.02	0.02
G2	RU	12.13	1.54	2.98	1.54	2.50	1.54	6.43	5.47
		13.29	2.13	3.68	2.13	3.19	2.13	7.32	6.27
	RA	8.14	1.62	4.08	1.62	3.33	1.62	5.79	4.08
		9.14	2.13	4.81	2.13	4.04	2.13	6.64	4.74
	PU (%)	0.11	0.04	0.05	0.04	0.05	0.04	0.08	0.08
		0.13	0.05	0.06	0.05	0.06	0.05	0.10	0.09
	PA (%)	0.07	0.03	0.04	0.03	0.03	0.03	0.05	0.05
		0.08	0.04	0.05	0.04	0.04	0.04	0.06	0.06
G3	RU	7.31	6.94	6.94	6.94	6.94	6.94	7.07	7.07
		7.45	7.08	7.07	7.08	7.08	7.08	7.21	7.21
	RA	7.25	7.10	7.12	7.09	7.11	7.10	7.18	7.17
		7.35	7.20	7.22	7.20	7.21	7.20	7.29	7.27
	PU (%)	33.11	33.14	32.99	33.14	33.06	33.14	33.05	33.12
		33.24	33.27	33.12	33.27	33.19	33.27	33.18	33.25
	PA (%)	17.43	17.44	17.39	17.44	17.42	17.44	17.41	17.44
		17.50	17.52	17.46	17.52	17.49	17.52	17.48	17.51

medRxiv preprint doi: https://doi.org/10.1101/2021.04.14.21255465; this version posted April 20, 2021. The copyright holder for this preprint (which was not certified by peer review) is the author/funder, who has granted medRxiv a license to display the preprint in perpetuity.
It is made available under a CC-BY 4.0 International license .

Table S7: We show (0.025, 0.975) percentiles of travel effects for different scenarios. For a given scenario, the upper value and lower value of each measurement are the 0.025 percentile value and the 0.975 percentile value, respectively. See Table S5 caption for more information.

		S-1	S-2	S-3	S-4	S-5	S-6	S-7	S-8
G	IA (%)	1.57	0.00	1.57	0.00	0.80	0.00	1.57	0.78
		1.67	0.00	1.68	0.00	0.85	0.00	1.68	0.83
	II (%)	2.68	0.00	2.68	0.01	1.36	0.00	2.68	1.32
		2.81	0.00	2.81	0.01	1.42	0.00	2.81	1.38
	IIQ (%)	2.68	0.00	0.00	0.01	0.00	0.00	1.32	1.32
		2.81	0.00	0.00	0.01	0.00	0.00	1.38	1.38
	Tc	100%	0%	100%	50%	50%	25%	100%	75%
	Te	100%	0%	5%	50%	3%	25%	52%	75%
G1	IA (%)	1.98	0.00	1.98	0.00	0.99	0.00	1.98	0.99
		2.09	0.00	2.09	0.01	1.04	0.00	2.10	1.05
	II (%)	3.10	0.00	3.10	0.01	1.57	0.00	3.10	1.54
		3.24	0.00	3.24	0.01	1.63	0.00	3.24	1.61
	IIQ (%)	3.10	0.00	0.00	0.01	0.00	0.00	1.53	1.54
		3.24	0.00	0.00	0.01	0.00	0.00	1.60	1.61
	Tc	100%	0%	100%	64%	50%	32%	100%	82%
	Te	100%	0%	5%	64%	3%	32%	52%	82%
G2	IA (%)	1.77	0.00	1.77	0.00	0.89	0.00	1.77	0.89
		1.89	0.00	1.89	0.01	0.95	0.00	1.90	0.95
	II (%)	3.02	0.00	3.02	0.01	1.52	0.00	3.02	1.51
		3.17	0.00	3.17	0.01	1.59	0.00	3.17	1.59
	IIQ (%)	3.02	0.00	0.00	0.01	0.00	0.00	1.51	1.51
		3.17	0.00	0.00	0.01	0.00	0.00	1.58	1.59
	Tc	100%	0%	100%	64%	50%	32%	100%	82%
	Te	100%	0%	5%	64%	3%	32%	52%	82%
G3	IA (%)	0.77	0.00	0.77	0.00	0.42	0.00	0.77	0.35
		0.82	0.00	0.82	0.00	0.45	0.00	0.82	0.37
	II (%)	1.57	0.00	1.57	0.01	0.85	0.00	1.57	0.72
		1.64	0.00	1.64	0.01	0.88	0.00	1.64	0.76
	IIQ (%)	1.57	0.00	0.00	0.01	0.00	0.00	0.72	0.72
		1.64	0.00	0.00	0.01	0.00	0.00	0.76	0.76
	Tc	100%	0%	100%	7%	50%	3%	100%	53%
	Te	100%	0%	5%	7%	3%	3%	52%	53%

medRxiv preprint doi: https://doi.org/10.1101/2021.04.14.21255465; this version posted April 20, 2021. The copyright holder for this preprint (which was not certified by peer review) is the author/funder, who has granted medRxiv a license to display the preprint in perpetuity.
It is made available under a CC-BY 4.0 International license .

References and Notes

1. D. J. Warne, *et al.*, *BMC Public Health* (2020).

2. D. T. Gillespie, *The Journal of Chemical Physics* **115**, 1716 (2001).

3. P. Marjoram, *et al.*, *PNAS* **100**, 15324 (2003).

4. S. A. Sisson, *et al.*, *PNAS* **104**, 1760 (2007).

5. T. Toni, *et al.*, *J. R. Soc. Interface* **6**, 187 (2009).

6. C. Drovandi, A. N. Pettitt, *Biometrics* **67**, 225 (2011).

7. https://github.com/anthonyebert/protoabc.

8. M. A. Beaumont, *et al.*, *Genetics* **162**, 2025 (2002).

9. K. Csilléry, *et al.*, *Trends in Ecology and Evolution Vol.25 No.7* **25**, 410 (2012).

10. D. Prangle, *Bayesian Analysis* **12**, 289 (2017).

11. B. Rahman, *et al.*, *Wiley* (2020).

12. O. Diekmann, *et al.*, *J. R. Soc. Interface* **7** (2010).
